# Comparing the succession of microbial communities throughout development in field and laboratory nests of the ambrosia beetle *Xyleborinus saxesenii*

**DOI:** 10.3389/fmicb.2023.1151208

**Published:** 2023-04-20

**Authors:** Janina M. C. Diehl, Alexander Keller, Peter H. W. Biedermann

**Affiliations:** ^1^Chair of Forest Entomology and Protection, Institute of Forestry, University of Freiburg, Freiburg im Breisgau, Germany; ^2^Insect-Fungus Interactions Research Group, Department of Animal Ecology and Tropical Biology, University of Würzburg, Würzburg, Germany; ^3^Faculty of Biology, Cellular and Organismic Networks, Ludwig-Maximilians-Universität München, Munich, Germany

**Keywords:** insect-fungus mutualism, microbial succession, symbiosis, metabarcoding, insect agriculture, field vs. laboratory studies, *Xyleborinus saxesenii*, ambrosia beetles

## Abstract

Some fungus-farming ambrosia beetles rely on multiple nutritional cultivars (Ascomycota: Ophiostomatales and/or yeasts) that seem to change in relative abundance over time. The succession of these fungi could benefit beetle hosts by optimal consumption of the substrate and extended longevity of the nest. However, abundances of fungal cultivars and other symbionts are poorly known and their culture-independent quantification over development has been studied in only a single species. Here, for the first time, we compared the diversity and succession of both fungal and bacterial communities of fungus gardens in the fruit-tree pinhole borer, *Xyleborinus saxesenii*, from field and laboratory nests over time. By amplicon sequencing of probed fungus gardens of both nest types at three development phases we showed an extreme reduction of diversity in both bacterial and fungal symbionts in laboratory nests. Furthermore, we observed a general transition from nutritional to non-beneficial fungal symbionts during beetle development. While one known nutritional mutualist, *Raffaelea canadensis*, was occurring more or less stable over time, the second mutualist *R. sulphurea* was dominating young nests and decreased in abundance at the expense of other secondary fungi. The quicker the succession proceeded, the slower offspring beetles developed, suggesting a negative role of these secondary symbionts. Finally, we found signs of transgenerational costs of late dispersal for daughters, possibly as early dispersers transmitted and started their own nests with less of the non-beneficial taxa. Future studies should focus on the functional roles of the few bacterial taxa that were present in both field and laboratory nests.

## 1. Introduction

Advanced fungus-farming for nourishment is an ecological feature that evolved only a few times in insects, namely one lineage of termites and several lineages of both ants and wood-boring weevils, also termed ambrosia beetles (Curculionidae: Scolytinae and Platypodinae) (Mueller et al., 2005; Biedermann and Vega, 2020). In all these insect systems fungal ectosymbionts are grown within social societies and are consumed as the major food-source. Despite major differences between farming practices, these farming insects have several features in common. All insect farmers inoculate plant substrate with mutualistic fungi, which are grown in so-called “fungus gardens.” These gardens are dominated by specific, nutritional fungi and protected by the insects from fungal competitors and pathogens by various means, comprising sequestration from the environment, active monitoring and behavioral and/or antibiotic treatment (e.g., Currie and Stuart, 2001; Fernández-Marín et al., 2015; Van Arnam et al., 2018; Nuotclà et al., 2019; Diehl et al., 2022; Schmidt et al., 2022). Importantly, the nutritional fungi are not depleted by the feeding insects, but the more individuals there are, the easier it is to maintain them (Biedermann and Rohlfs, 2017). Thus, fungus gardens can provide food within one nest for multiple generations of insects (Mueller et al., 2005).

Despite some common features in the fungiculture of ants, termites and weevils, there are also major differences regarding the substrate supply for the fungi and the homogeneity of the nest environment. In contrast to ants and termites that actively forage on a diversity of substrates (e.g., leaves, elytra) for consecutive provisioning of their fungi (e.g., Wisselink et al., 2020; Römer et al., 2022), fungus farming weevils live and breed inside the wood-substrate they use for growing fungi (Kirkendall et al., 2015; Birkemoe et al., 2018). This has major impacts on the substrate conditions the fungus gardens are exposed to and the abundance and diversity of microbial competitors and pathogens inside fungus gardens. First, substrate quality for the nutritional fungi quickly deteriorates in ambrosia beetles because it is not replaced and essential nutrients are consumed by the fungi over time (Nuotclà et al., 2021). Beetles can counteract this effect to a limited degree by recycling their feces (Abrahamson and Norris, 1970; De Fine Licht and Biedermann, 2012) and expansion of their tunnel systems inside the wood, but latter is strongly constrained by intraspecific competition and general degradation of the wood (which is typically colonized shortly after the death of the host tree) (Kirkendall et al., 2015; Birkemoe et al., 2018). Second, while fungus gardens of ants and termites are very exposed to microorganisms that are constantly brought in with the new plant substrate and the surrounding soil (Pagnocca et al., 2012; Estrada et al., 2014; Chiri et al., 2020, 2021; Chen et al., 2021), gardens of ambrosia beetles are only exposed to microorganisms (i) already present in the wood at colonization (i.e., endosymbionts), (ii) initially brought in with the nest-founding beetle(s), or (iii) entering gardens from the surrounding wood over time. In relation, this massively reduces the exposure of ambrosia beetles to microbial threats and also explains their comparatively less advanced techniques of pathogen control (Mighell and Van Bael, 2016; Diehl et al., 2022).

As outlined above termites and ants grow a single dominant fungal species over multiple generations using a diversity of substrates and maintenance strategies (Shinzato et al., 2005; Mehdiabadi et al., 2006; Mueller et al., 2010). By contrast, some ambrosia beetle species in the genera *Xyleborus* seem to be able to develop on a variety of nutritional cultivars (Menocal et al., 2023). Moreover, in *Xyleborus* and *Xyleborinus* (Scolytinae) stable relationships with one or more co-occurring nutritional *Raffaelea* species (Ascomycota: Ophiostomatales) and/or yeasts (Ascomycota) exist (Cruz et al., 2019; Ibarra-Juarez et al., 2020; Nuotclà et al., 2021; Diehl et al., 2022). Signs for succession of these putative mutualists may indicate optimal consumption of the substrate, through variation in enzymatic capabilities of the fungi (De Fine Licht and Biedermann, 2012; Ibarra-Juarez et al., 2020). However, abundances of fungal symbionts are poorly studied and despite a diversity of >3,000 species of ambrosia beetles, culture-independent quantification of symbiont communities over beetle development has been applied in only a single species (*Xyleborus affinis*; Ibarra-Juarez et al., 2020).

*Xyleborus* and *Xyleborinus* ambrosia beetles construct their nests as tunnel systems (termed “galleries”) in the xylem of trees (typically weakened or recently dead) (Beaver, 1989). *Raffaelea* fungi, and in some cases possibly ascomycete yeasts, serve as exclusive food source and provide their hosts with essential vitamins, amino acids and sterols (Kok et al., 1970; Beaver, 1989; Saucedo-Carabez et al., 2018; Cruz et al., 2019; Ibarra-Juarez et al., 2020). These typically species-specific food fungi are taken up by adult females inside their guts and/or oral or elytral mycetangia (=selective spore-carrying organs) within their natal nest, before they disperse and establish their own nest and fungus garden (Francke-Grosmann, 1956, 1967, 1975; Mayers et al., 2022). Other unspecific Ophiostomatales fungi, yeasts and various groups of filamentous fungal saprophytes and plant pathogens [Hypocreales (e.g., *Fusarium, Beauveria*), Eurotiales (e.g., *Penicillium, Aspergillus, Paecilomyces, Talaromyces*), Botryosphaeriales (e.g., *Diplodia*), Dothideales (e.g., *Aureobasidium*), Pleosporales (e.g., *Alternaria*), and Cladosporiales (e.g., *Cladosporium*)] are typically co-transmitted from natal nests in low abundances, probably mostly unintentionally on beetle surfaces (Batra and Batra, 1979; Biedermann et al., 2013; Kostovcik et al., 2015; Saucedo-Carabez et al., 2018; Cruz et al., 2019; Biedermann, 2020; Ibarra-Juarez et al., 2020; Nuotclà et al., 2021). Apart from some obvious antagonists or pathogens (e.g., *Aspergillus, Beauveria*) the functional roles of these fungi have not been determined, but given their mostly infrequent occurrence they are regarded non-beneficial for beetle fitness. The fact that these fungal antagonists increase in abundance the older a nest gets (e.g., Biedermann, 2020; Ibarra-Juarez et al., 2020) may, however, affect the type and quantity of fungal antagonists transmitted by dispersing daughter females when they leave their natal nest. The timing of daughter dispersal may thus potentially have transgenerational effects on beetle fitness, but this has not yet been determined.

Recent experimental evidence suggests that the presence of the fruit-tree pinhole borer, *Xyleborinus saxesenii* (Ratzeburg) on its fungus garden promotes the dominance of *Raffaelea* nutritional mutualists over antagonists (Diehl et al., 2022). Similarly, removal of fungal pathogens has been repeatedly observed in other *Xyleborus* and *Xyleborinus* ambrosia beetles (Kingsolver and Norris, 1977; Biedermann et al., 2013; Nuotclà et al., 2019; Biedermann, 2020). However, the mechanisms underlying this selective exclusion and promotion of nutritional fungi are unknown. It is possible that bacteria are playing a role in this defense (Grubbs et al., 2020), similar to specific defenses by bacteria in fungus-farming ants and termites (e.g., Van Arnam et al., 2018). Although the functional role of bacteria in ambrosia beetle communities has not been experimentally determined, similar bacterial groups dominate in all fungus-farming insect groups (Aylward et al., 2014). In ambrosia beetles bacterial taxa mainly belong to the classes of Alpha- (e.g., *Ochrobactrum, Phyllobacterium, Sphingomonas*), Beta- (e.g., *Burkholderia*) and Gammaproteobacteria (e.g., *Pseudomonas, Pseudoxanthomonas, Erwinia, Stenotrophomonas, Pantoea*), Sphingobacteria (e.g., *Pedobacter, Olivibacter, Sphingobacterium*), Actinobacteria (e.g., *Streptomyces, Microbacterium*), Flavobacteria (e.g., *Chryseobacterium*), Bacilli (e.g., *Staphylococcus, Bacillus*), and Chitinophagia (e.g., *Niabella*) (Fabig, 2011; Aylward et al., 2014; Ibarra-Juarez et al., 2020; Nuotclà et al., 2021; Nones et al., 2022). In *X. affinis*, cellular pathway analyses suggest that its bacterial symbionts contribute in wood degradation, nitrogen fixation and nutritional provisioning (Ibarra-Juarez et al., 2020).

Most studies on fungus-garden communities of insects are either done with material collected in the field or from laboratory nests. Both have their benefits and disadvantages. While laboratory rearing has little effect on the traits of some invertebrates (Kölliker-Ott et al., 2003; Jong et al., 2017), the traits of other species no longer reflect those of natural populations (Meats et al., 2004; Liedo et al., 2007). Field studies offer more realistic conditions, but experimental manipulations and high sample sizes are often possible only in the laboratory (Calisi and Bentley, 2009). The development of laboratory rearing for ambrosia beetles was a breakthrough for research on these species, especially regarding behavioral studies, but also for studying the effects of microbial manipulations, because their wood-tunneling behavior did not allow observations of ambrosia beetles in the field (Saunders and Knoke, 1967; Biedermann et al., 2009). However, so far, we have no knowledge if and how much fungus-garden microbial communities and their succession are influenced by the artificial rearing substrate. Due to the addition of sugars, fats and proteins, the latter is more nutrient rich and lower in plant secondary metabolites (phenolics and terpenoids, which are destroyed by autoclaving) compared to wood. Nevertheless, brood sizes between field and laboratory are comparable even though development is much faster in the laboratory, probably due to higher and stable temperatures (Biedermann et al., 2009).

This is the first attempt to compare the diversity and succession of both fungal and bacterial communities in ambrosia beetle fungus gardens from field and laboratory nests (i.e., in artificial media) over time. All gardens are collected from nests of the fruit-tree pinhole borer, *X. saxesenii*, out of the same population, at the same time and within substrate of the same tree species (beech trees in the field vs. beech sawdust in the lab). In both field and lab, we probed fungus garden communities at three development phases of nests (immature brood vs. immature and adult brood vs. only adult brood present); laboratory nests allowed us to collect additional information on the speed of beetle development in relation to symbionts and transgenerational effects of early or late dispersal of females from their natal nests on their own fungus garden communities later on. Using these methods, we tested the following predictions. Since rearing medium is autoclaved, we expect laboratory fungus gardens to host mostly the vertically transmitted bacterial and fungal symbionts required for nutrition and defense. Under natural conditions, a much more diverse and unstable community may be present, including the beneficial symbionts, but also other environmentally acquired microbial associates. This could result in lower abundances of the nutritional fungi in the field, due to competition. Finally, development speed may increase with the presence of more nutritional fungi, and the later dispersal of daughters from nests that harbor higher abundances of non-mutualists, may led to less beneficial symbiont communities when they found their own gardens later on.

## 2. Materials and methods

### 2.1. Field collection of nests and beetle laboratory rearing

In this study all beetles and nests (=“galleries”) collected for both field and lab sampling originated from a population of *X. saxesenii* in the Steinbachtal near Wuerzburg (49.767500, 9.896770/49°46′03.0″N 9°53′48.4″E), Germany. We marked four recently dead and wind-thrown beech tree logs (*Fagus sylvatica*) that were colonized by *X. saxesenii* in spring 2018. The examination of field nests is destructive, so we repeatedly went there between July to October 2018 to collect field nests at different developmental stages.

From the same beetle population, females for laboratory rearing were collected in the field by using ethanol baited traps (70% EtOH) during their dispersal flight in May 2018. After rinsing females first with 70% EtOH and then tap water, they were dried on cosmetic towel and individually put into transparent plastic tubes filled with–previously prepared–sterile artificial medium [“standard media” after (Biedermann et al., 2009)]. These wild-caught female foundresses build the parental generation (=F0) and were bred under standard conditions (20°C, complete darkness). They immediately start tunneling and 4–7 days later fungal symbionts start to cover the tunnel walls (i.e., “fungus garden”) (Biedermann et al., 2009). About 40 days later dispersal of sib-mated, adult daughters starts and 150 of these, from 18 different nests, were collected and after sterilization with 70% EtOH (see above), again introduced onto new rearing medium. This F1 generation of lab-born foundresses was then used for the following detailed examinations of symbiont communities and development.

### 2.2. Fungus garden sampling

#### 2.2.1. Field nest classification and fungus garden sampling

**FIGURE 1:**
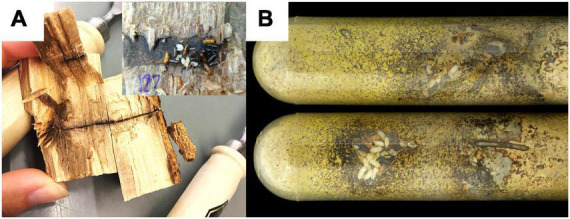
Two types of nests sampled for the study. **(A)** Two opened *X. saxesenii* nests from *F. sylvatica* logs (field nests). One showing the long entrance tunnel, the other shows the brood chamber in focus. **(B)** Laboratory nests in artificial medium. Yellow coloration of the medium due to growth of the nutritional mutualist *R. sulphurea*. White individuals are larvae or pupae, light-brown ones are teneral females and black ones are fully-sclerotized adult females.

In the field we sampled fungus gardens out of 30 nests. Log parts were brought to the laboratory and nests were opened using a cleaver, chisel and hammer (Figure 1A). We classified the phase of development in (i) nests with eggs and larvae, (ii) nests with larvae, pupae and adult offspring, and (iii) nests with only adults present (Table 1). After aseptic removal of all individuals, fungus gardens of these nests were sampled by slicing off thin layers of the nest walls (near the center of the nest) with a flame-sterilized scalpel. These slices were aseptically stored in 1.5 ml Eppendorf tubes at −20°C until DNA extraction.

**TABLE 1:** Classification of field and laboratory nests by their phase and speed of development.

Development	*N*_Field_	Development speed*	*N*_Lab_
Phase 1 larvae 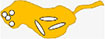	13	Fast (7–9 days)	6
		Medium (10–12 days)	28
		Slow (13–31 days)	16
Phase 2 larvae–pupae–adults 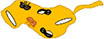	13	Fast (25–26 days)	11
		Medium (27–31 days)	25
		Slow (32–43 days)	14
Phase 3 adults 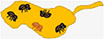	4	Fast (35–41 days)	16
		Medium (42–47 days)	20
		Slow (48–76 days)	14

Numbers of nests (*N*) per classification are given.

*Categorization of the developmental speed was only applied to laboratory nests.

#### 2.2.2. Laboratory nest classification and fungus garden sampling

Laboratory rearing has the advantage that nest development can be directly monitored through the transparent walls of the rearing tubes (Figure 1B). Therefore, we were not only able to record the “developmental phase” of the nest (see *field sampling*), but could distinguish also “fast,” “medium,” and “slow” developing beetle nests (Table 1). Furthermore, we also had information about the timing of dispersal of the F1 foundresses from their natal nests–here classified as “early,” “middle,” or “late” disperser. The timing of dispersal may have an influence on the symbiont community dispersed, as there is a succession of symbiont communities in nests over time (e.g., Ibarra-Juarez et al., 2020). For fungus garden sampling, we destructively dissected nests by first knocking out the solid rearing medium (containing the nest) out of the rearing tube, removal of all individuals and then collecting pieces of the nest walls from the nest center under the sterile bench with a flame-sterilized sharp spoon (mean weight of pieces ± SD = 96.47 mg ± 34.34). Fungus garden samples were aseptically stored in 1.5 ml Eppendorf tubes at −20°C until DNA extraction.

### 2.3. DNA extraction and library preparation

DNA of all samples was extracted using the ZymoBIOMICS DNA Miniprep Kit (Zymo Research Europa GmbH, Freiburg, Germany) in accordance with the manufacturer’s instructions and additional pre-processing steps with a ceramic bead and a mixer mill (Retsch GmbH, Haan, Germany), followed by an additional step with glass beads (0.1 mm and 0.5 mm) vortexed on a Vortex Genie 2 [see Supporting Material in Nuotclà et al. (2021)]. The isolated DNA samples were stored at −20°C until the final amplification and sequencing.

PCRs and library preparation were performed as in previous projects (see Nuotclà et al., 2021; Diehl et al., 2022) in triplicate reactions (each 10 μl) in order to avoid PCR bias. Bacteria 16S-rRNA gene libraries were constructed using the dual-indexing strategy described in Kozich et al. (2013) using the 515f and 806r primers that amplify amplicon sequences of a mean merged length of 246.17 bp_*field*_/237.56 bp_*lab*_, encompassing the full V4 region (modified from Caporaso et al., 2011). Conditions for the PCR were as follows: initial denaturation at 95°C for 4 min, 30 cycles of denaturation at 95°C for 40 s, annealing at 55°C for 30 s and elongation at 72°C for 1 min, followed by a final extension step at 72°C for 5 min.

Fungal LSU (28S) rRNA gene libraries (mean merged length of 280.67 bp_*field*_/276.63 bp_*lab*)_ were constructed similarly from the same samples by using the dual-index primers of LIC15R and nu-LSU-355-3′ (Nuotclà et al., 2021) to amplify the large subunit (LSU) region. Conditions for the PCR were as follows: initial denaturation at 98°C for 30 s, 35 cycles of denaturation at 98°C for 30 s, annealing at 55°C for 30 s and elongation at 72°C for 15 s, followed by a final extension step at 72°C for 10 min. Sample-specific labeling for both bacterial and fungal DNA was achieved by assigning each sample to a different forward/reverse index combination.

After both PCRs, triplicate reactions of each sample were combined per marker and further processed as described in Kozich et al. (2013), including between-sample normalization using the SequalPrep™ Normalization Plate Kit (Invitrogen GmbH, Darmstadt, Germany) and pooling of 96 samples. The pools were cleaned-up with the AMPure Beads Purification (Agilent Technologies, Inc., Santa Clara, CA, USA) and quality controlled using a Bioanalyzer High Sensitivity DNA Chip (Agilent Technologies, Santa Clara, CA, USA) and quantified with the dsDNA High Sensitivity Assay (Life Technologies GmbH, Darmstadt, Germany). Afterward, pools were combined to a single library pool containing 384 samples in total. This library was diluted to 8 p.m., denatured and spiked with 5% PhiX Control Kit v3 (Illumina Inc., San Diego, CA, USA) according to the Sample Preparation Guide (Illumina Inc., 2013). Sequencing was performed on an Illumina MiSeq using 2 × 250 cycles v2 chemistry (Illumina Inc., San Diego, CA, USA). Each marker was processed on a separate chip. We used our custom scripts to process the reads and assign the taxonomy. See Supplements in Diehl et al. (2022) and GitHub Repository for further methods on sequencing controls and details on bioinformatics processing.

### 2.4. Statistical analysis of molecular data

All statistical analyses and visualization of the sequence output were performed in RStudio (Version 1.4.1106) with R version 4.0.5 (R Core Team, 2021) using the phyloseq package (McMurdie and Holmes, 2013); see GitHub repository for information on the bioinformatic processing and R-script.

#### 2.4.1. Field samples data preparation

After excluding control samples, 30 out of the 36 field samples were left for further analyses. Further removal of Chloroplast genes, ASVs (amplicon sequence variants) that were only identified to domain level and running “decontam” (Davis et al., 2018) for the 16S field data, left an average of 24,383 reads/sample for downstream analysis (range from 13,116 to 42,604). In total, 242 bacterial ASVs ran into the analysis. Bacterial composition was studied up to the genus level and their relative abundance (RA). For the LSU, only ASVs that were not further identified than to domain level and control samples were excluded and left an average of 24,702 reads/sample for downstream analysis (range from 4,830 to 40,839). In total, 451 fungal ASVs ran into analysis. Fungal composition was studied up to the species level and their relative abundance (RA).

#### 2.4.2. Laboratory samples data preparation

A total of 82 out of the 151 samples (excluding 20 controls) showed infection with the endosymbiont *Wolbachia* or had low read numbers (≤500 reads). The ASVs identifying *Wolbachia* were excluded from further analyses, since insect related infection was not in the focus of our research on fungus garden material. It is worth mentioning, however, that *Wolbachia* has been present in several analyses of laboratory nests by now (this study and Nuotclà et al., 2021; Diehl et al., 2022), whereas material of field nests never contained *Wolbachia*.

The further removal of ASVs and samples describes earlier left an average of 16,899 reads/sample for downstream analysis (range from 2,628 to 48,835). In total, 166 bacterial ASVs ran into the analysis. For the LSU, we ended up with an average of 21,245 reads/sample for downstream analysis (range from 2,190 to 55,630). In total, 246 fungal ASVs ran into the analysis. Two out of the 150 samples (excluding 23 controls) had low read numbers (≤500 reads) and were therefore excluded from further analyses.

#### 2.4.3. Rarefaction of sequence reads for the analysis of alpha diversity

For the analysis of the alpha diversity we rarefied the sequence reads of all samples depending on the quality of the datasets (Supplementary Figure 1). For the field samples we decided to rarefy to a total of 10,000 reads/sample for the bacterial community and 4,000 reads/sample for the fungal community. Rarefaction removed two ASVs from the bacterial and 32 ASVs from the fungal dataset. The laboratory samples were rarefied to a total of 2,500 reads/sample for the bacterial community and 2,000 reads/sample for the fungal community. Rarefaction removed 60 ASVs from the bacterial and 32 ASVs from the fungal dataset.

#### 2.4.4. Analysis of alpha diversity

We applied the chi-square tests to both the total number of ASVs in field and laboratory community data to test whether the number of ASVs was significantly different. To investigate the microbial diversity and richness of fungus gardens, we calculated the observed richness (OR) and Shannon’s diversity index (SDI) (“microbiome” package: Lahti and Shetty, 2017). For both measures we ran a generalized linear mixed-effects model (GLMM) with “tree origin” and “lineage” (F1 females originated from different F0 families) as random variable, assuming a normal distribution (“glmmTMB” package: Brooks et al., 2017) to test for the influence of the “developmental phase” on the microbial community. Previous analyses (Diehl et al., 2022) showed strong heritable effects of lineage and tree identity on symbiont communities in *X. saxesenii*. For laboratory samples we further ran linear mixed models (LMMs) to test the additional influence of dispersal time of the foundress (“*early*” vs. “*middle*” vs. “*late*”) and development speed of the nest (“*fast*” vs. “*medium*” vs. “*slow*”) on the microbial community. We implemented mixed models using the “lme” function (“nlme” package: Pinheiro et al., 2021) and used the “transformTukey” function (rcompanion package; Mangiafico, 2022) to find the power transformation that brought the alpha diversity effects closest to a normal distribution.

All LMMs were initially fitted with all interaction terms. Best-fitting models were selected by the following procedure: First, we used the Akaike information criterion (AIC) to select an appropriate variance structure (using the weights-argument in the “lme” function), when residual plots indicated a deviation from homogeneity (Zuur et al., 2009). Second, we simplified the fixed component by dropping non-significant interaction terms (*p* > 0.05). In a last step, we used–if necessary–the AIC to select the appropriate transformation method to produce a more-normally distributed vector (using squared- or Tukey-transformed response variables with the “transformTukey” function of the “rcompanion” package, Mangiafico, 2022).

We obtained the *p*-values of effects in these models using the ANOVA function (which uses type II sums of squares by default; Fox and Weisberg, 2019). Significant models were further analyzed using a pairwise *post-hoc* test (Tukey method; “emmeans” package: Lenth, 2022) to identify differences between groups. The package “ggplot2” (Wickham, 2016) was used to build the figures for alpha diversity.

#### 2.4.5. Analysis of beta diversity

To visualize differences in microbial composition (beta diversity), we applied non-metric multidimensional scaling (NMDS, “phyloseq” package: McMurdie and Holmes, 2013) on Bray Curtis dissimilarities derived from proportion transformed data, which consider presence/absence as well as abundance of ASVs (Clarke et al., 2006). To compare the microbial communities between the “*developmental phase*” and the “*tree origin*” for the field data, we performed a permutational ANOVA test (PERMANOVA) on Bray-Curtis distance matrices of the proportion data using the R package “vegan” (Oksanen et al., 2020). Significant results were examined in more detail with a pairwise comparison of adjusted *p*-values (“pairwiseAdonis” package: Martinez Arbizu, 2020). The homogeneity of multivariate dispersions was tested with a permutation test [“vegan” package: (Oksanen et al., 2020)] applied on each the “*development phase*” and “*tree origin.*” Since we were able to collect more qualitative data in the laboratory bred nests we tested in the PERMANOVA the variables “*development phase*,” “*development speed*,” and “*dispersal time*” nested in the variable “*family lineage*” to compare the communities in relation to the groups. With heatmaps of the microbial composition [“microbiome” package: (Lahti and Shetty, 2017)], we concluded the overview of the beta diversity.

#### 2.4.6. Closer look on major fungal taxa of field and laboratory galleries

We ran another set of LMMs on subsets of the most abundant fungi to test whether relative abundances of these specific taxa differed between the development phases. For example, we compared the relative abundances of the two ambrosia fungi, *Raffaelea sulphurea* (aka *Dryadomyces sulphureus*) and *R. canadensis*, and the commensal fungus *Chaetomium globosum* in laboratory galleries. Here, the relative abundances (RA) of the fungi were set as the response variables, and the phases and speed of development as well as the timing of maternal dispersal served as explanatory variables. The family lineage was included as a random factor. Most common taxa chosen for the field galleries were, next to the ambrosia fungi, *Graphium sp.* and Sordariomycetes (unknown). RA of the taxa were set as response and development phase as explanatory variable. Tree origin of sampled galleries was included as a random factor. The analysis followed the same procedure of fitting and selection as in the previous LMMs.

#### 2.4.7. Analysis of correlation between bacterial and fungal communities

To investigate the correlation between the bacterial and fungal communities in our field and laboratory samples, we employed the Bray-Curtis method using the vegdist() function from the “vegan” package (Oksanen et al., 2020) to create matrices of dissimilarity indices based on the relative abundances of each community. To ensure a fair comparison, we made subsets of our laboratory dataset to 87 matching samples for both communities. The correlation was then determined using the Mantel statistic (also from the “vegan” package) with 999 permutations.

#### 2.4.8. Additional packages used

The packages “fitdistrplus” (Delignette-Muller and Dutang, 2015), “performance” (Lüdecke et al., 2021), and “Dharma” (Hartig, 2020) were applied in testing for the best distribution, as well as model fit. “ggplot2” (Wickham, 2016), “scales” (Wickham and Seidel, 2022), and “ggpubr” (Kassambara, 2020), “ggrepel” (Slowikowski, 2021), “lattice” (Sarkar, 2008), and “cowplot” (Wilke, 2020) were used to build the figures. “dplyr” (Wickham et al., 2022) was used for data manipulation.

## 3. Results

### 3.1. Bacterial diversity of fungus gardens in field and laboratory nests

**FIGURE 2:**
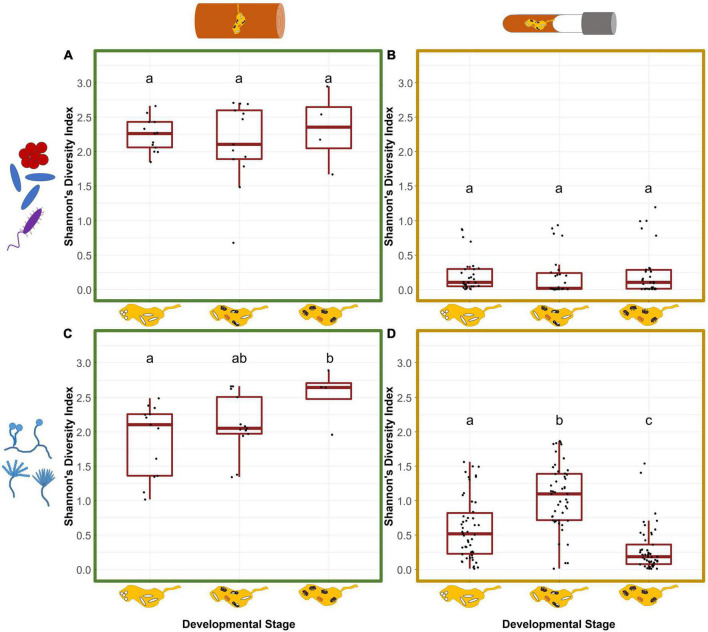
Symbiont diversity of field and laboratory fungus gardens in relation to the nest development phases of *X. saxesenii* nests. Box-plots for Shannon’s diversity index of bacterial **(A,B)** and fungal **(C,D)** communities of fungus gardens in field **(A,C)** and laboratory **(B,D)** nests. Lowercase letters indicate significant differences between groups (Tukey’s HSD test: *p* < 0.05).

In general, both diversity and richness of bacteria was much higher in field (242 ASVs) than laboratory (155 ASVs) nests (chi-square test: χ^2^ = 19.07, *df* = 1, *p* = < 0.001). In both groups, bacterial diversity did not change over the course of nest development (SDI_*field*_: GLMM: χ^2^ = 1.48, *p* = 0.477; SDI_*lab*_: LMM: χ^2^ = 4.10, *p* = 0.129; Figure 2) and effects of development phase on richness were apparent only in lab nests (OR_*field*_: χ^2^ = 0.113, *p* = 0.945; OR_*lab*_: χ^2^ = 15.94, *p* = < 0.001; Supplementary Figure 5). Development phases of field and laboratory nests slightly affected bacterial beta diversity in fungus gardens (see details in Supplementary material). There was no effect of timing of foundress dispersal on bacterial beta diversity of lab nests (PERMANOVA: *R*^2^ = 0.043, *F* = 1.95, *p* = 0.652). The tree the field nests originated from, had a strong effect on the bacterial community composition (*R*^2^ = 0.236, *F* = 2.85, *p* = 0.001), however.

### 3.2. Fungal diversity of fungus gardens in field and laboratory nests

Similar to the observation for bacteria, the diversity and richness of the fungal community was much higher in field (451 ASVs) than laboratory (246 ASVs) nests (chi-square test: χ^2^ = 60.29, *df* = 1, *p* = < 0.001). Fungal diversity increased over the course of nest development (SDI_*field*_: GLMM: χ^2^ = 6.65, *p* = 0.036; SDI_*lab*_: LMM: χ^2^ = 133.2, *p* = < 0.001; Figure 2). While field fungus gardens had the highest diversity when only adults were present (phase1 vs. 3: *t* = −2.55, *p* = 0.044), lab-garden fungal diversity peaked earlier during the presence of immature and adult offspring (phase 1 vs. 2: *t* = −5.86, *p* = < 0.001) and had the lowest diversity in later stages when only adults were present (phase 1 vs. 3/phase 2 vs. 3; *p* < 0.001, Figure 2). Slow-developing nests tended to have lower SDI than medium (*t* = 2.42, *p* = 0.045). This was not the case for fast developing nests which showed neither a difference compared to medium nor to the slow developing nests (“fast-medium” *p* = 0.697, “fast-slow” *p* = 0.247) (Supplementary Figure 4). The factor development only affected fungal richness of laboratory nests, with the highest OR during the presence of immature and adult offspring (OR_*field*_: χ^2^ = 0.372, *p* = 0.830; OR_*lab*_: χ^2^ = 15.41, *p* = < 0.001; Supplementary Figure 5).

For the fungal beta diversity of field gardens, there was a stronger effect of development phase (PERMANOVA: *R*^2^ = 0.148, *F* = 2.61, *p* = 0.007) than for the tree of origin (*R*^2^ = 0.175, *F* = 2.06, *p* = 0.028). Both phase and speed of development influenced fungal beta diversity of lab gardens (PERMANOVA: *R*^2^ = 0.056, *p* = 0.05) (for more details see Supplementary material).

### 3.3. The major microbial community of fungus gardens in field and laboratory nests

**FIGURE 3:**
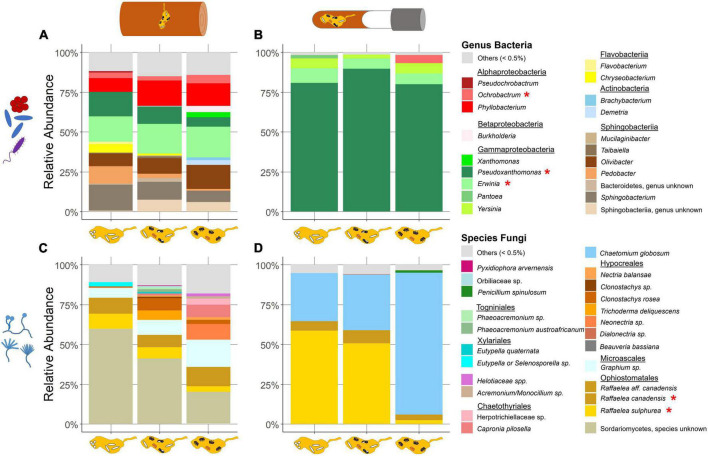
Relative abundance of symbiont taxa in field and laboratory fungus gardens in relation to the development phases of *X. saxesenii* nests. Community of bacterial genera **(A,B)** and fungal species **(C,D)** in fungus gardens of field **(A,C)** and laboratory **(B,D)** nests with a relative abundance of at least 0.5% (all else is combined in “others”). Taxa marked with (*) were found in both field and laboratory communities.

Altogether 13 bacterial classes were detected across field samples. Among these, Actinobacteria, Chitinophagia, Flavobacteriia, Sphingobacteriia, Alphaproteobacteria, Betaproteobacteria, and Gammaproteobacteria were most abundant (>0.5% mean RA) and accounted for approximately 90% of total sequences (Figure 3A; Supplementary Table 2). Gammaproteobacteria comprised ASVs of the genera *Pseudoxanthomonas* (mean + s.d. = 12.43% ± 7.25 RA), *Erwinia* (9% ± 13.49) and *Xanthomonas* (0.7% ± 2.58). Betaproteobacteria were mostly represented by *Burkholderia* (0.76% ± 3.03). Alphaproteobacteria were dominated by *Phyllobacterium* (13.14% ± 10.66), *Ochrobactrum* (4.11% ± 5.15), *Pseudochrobactrum* (0.56% ± 2.15), *Mesorhizobium* (0.53% ± 0.72), and *Roseomonas* (0.51% ± 0.95). Four ASVs of Sphingobacteriia appeared frequently in the nests. Most abundant were *Sphingobacterium* (13.56% ± 12.83) and *Olivibacter* (10.62% ± 10.41), followed by *Pedobacter* (6.8% ± 8.23) and an unknown Sphingobacteriia (4.55% ± 9.59). Chitinophagia were represented by the genus *Taibaiella* (1.36% ± 2.36) an Actinobacteria by *Demetria* (0.52% ± 2.3). Another more abundant class included the Flavobacteriia with the genera *Chryseobacterium* (3.54% ± 5.09) and *Flavobacterium* (0.98% ± 2.55). Lastly, an ASV of the phylum Bacteroidetes (not specified) was found in almost half of the nests (1.42% ± 6.04). Bacilli, Cytophagia, Deinococci, Thermoleophilia, and Verrucomicrobia were detected in relative abundances less than 0.5% of mean total reads.

Laboratory samples covered 16 classes but only four ASVs with higher relative abundance (Figure 3B; Supplementary Table 3). The most dominant class here was the Gammaproteobacteria with its highly abundant *Pseudoxanthomonas* (83.32% ± 30.65) and *Erwinia* (7.56% ± 21.72), *Pantoea* (0.81% ± 7.35) and *Yersinia* (5.18% ± 20.2). Alphaproteobacteria were almost exclusively represented by *Ochrobactrum* (1.64% ± 11.57). We also found Actinobacteria in an abundance of over 0.5% mean RA. Acidobacteria, Bacilli, Bacteroidia, Betaproteobacteria, Chitinophagia, Clostridia, Deinococci, Deltaproteobacteria, Flavobacteriia, Mollicutes, Negativicutes, Planctomycetacia and Sphingobacteriia were observed in relative abundances less than 0.5% of mean total reads.

The analyses of the field dataset yielded 15 fungal orders. Among these, Ophiostomatales, Microascales, Xylariales, Chartothyriales, Hypocreales, and Togniniales were most abundant (>0.5% mean RA; Figure 3C; Supplementary Table 2). The highest relative abundance came from an unknown Sordariomycetes (46.6% ± 24.13). The most abundant order was the Ophiostomatales with the ambrosia fungi *R. sulphurea* (9.33% ± 8.0), *R. canadensis* (10.34% ± 12.13) and *R. aff. canadensis* (0.78% ± 1.34). Microascales were represented by a *Graphium sp*. (9.95% ± 11.94). The order Hypocreales (11.87% ± 16.72) included two ASVs of the genus *Clonostachys*, *Nectria balansae*, a *Neonectria sp.*, and *Trichoderma deliquescens* (Supplementary Table 2). Other ASVs with a relative abundance greater than 0.5% total mean RA were *Phaeoacremonium sp.* (Togniniales; 1.75% ± 5.20), a *Diatrypaceae* (unknown) (Xylariales; 1.24% ± 4.47) and a Herpotrichiellaceae (unknown) (Chaetothyriales, 0.72% ± 2.84) (Supplementary Table 2). Moreover, additional fungi in the orders Eurotiales, Sordariales, Capnodiales, Helotiales, Coniochaetales, Saccharomycetales, Pyxidiophorales, Pleosporales, and Orbiliales were successfully amplified, but below the threshold of 0.5% total mean RA.

Less diversity was found in the laboratory dataset. Here, we detected 11 fungal orders, but only three higher abundant taxa (Figure 3D; Supplementary Table 3). Again, the two ambrosia fungi, *R. sulphurea* (38.61% ± 38.11) and *R. canadensis* (6.67% ± 15.49) were identified. Further, *C. globosum* (Sordariales; 52.16% ± 41.33) was detected in all nests and about a third of the nests contained some Eurotiales (0.58% ± 5.65) (Supplementary Table 3). Additional fungi in the orders Capnodiales, Chaetothyriales, Dothideales, Saccharomycetales, Hypocreales, Pleosporales, Microascales, and Togniniales were exposed, but below the threshold of 0.5% total mean RA.

In the sequence output of the positive controls we were only able to detect four out of the present six fungal genera. While we got sequence results from some taxa in the Saccharomycetales order in the experimental samples, the primers failed to amplify the yeasts *Pichia* sp. and *Candida* sp. in the mock and Zymo control (Supplementary Figures 2C, E, 3C, E).

### 3.4. The abundance of most common taxa within fungus gardens of field and laboratory nests

**FIGURE 4:**
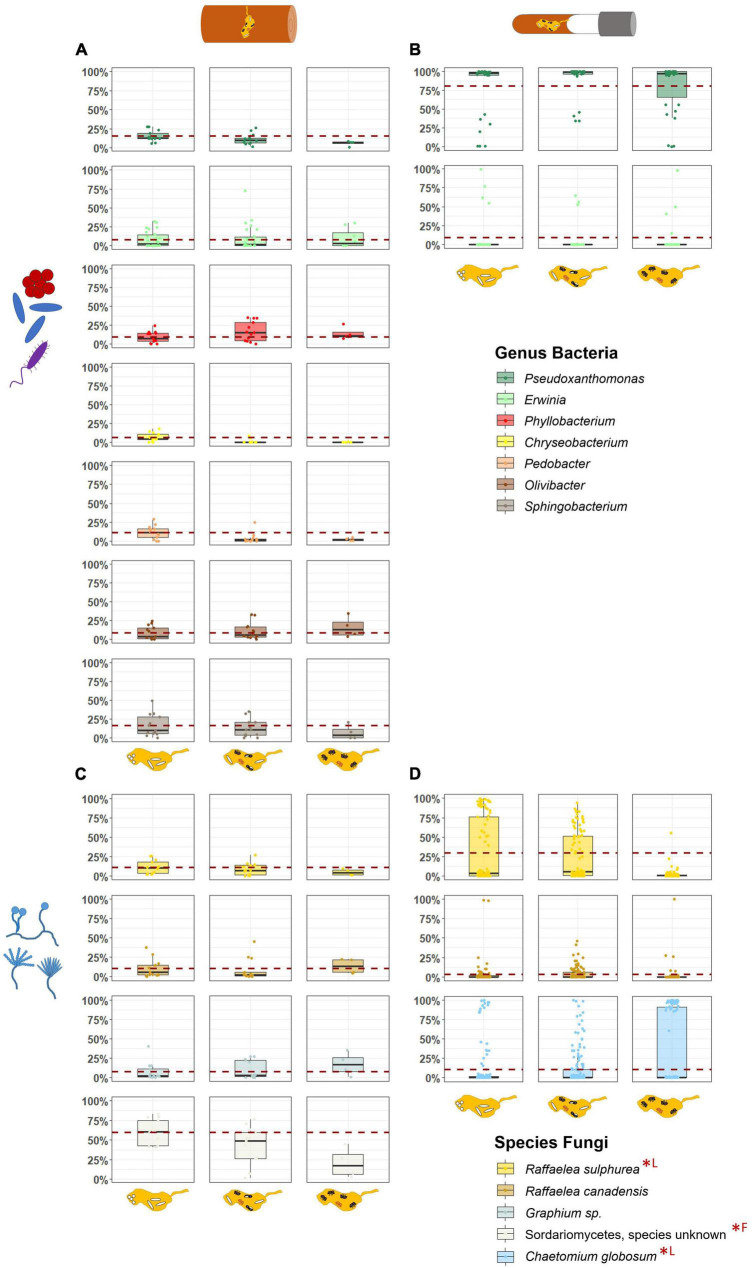
Comparison of the relative abundances of most important symbionts in field and laboratory fungus gardens in relation to the development phases of *X. saxesenii* nests. Box-plots with the relative abundance of dominant bacterial genera **(A,B)** and fungal species **(C,D)** in fungus gardens of field **(A,C)** and laboratory **(B,D)** nests. Dashed red line represents mean relative abundance of the first developmental phase. Statistical differences (*p* < 0.05) of abundances between development phases are marked with (*; *F* = field nests, *L* = laboratory nests).

Only one bacterial ASV each in the genera *Pseudoxanthomonas* and *Erwinia*, and the two ambrosia fungi, *Raffaelea sulphurea* and *R. canadensis*, occurred in considerable abundance both in field and laboratory nests (Figure 4). Fungus gardens from the field additionally harbored several other bacterial taxa and two more fungi, a *Graphium* sp. and an unknown Sordariomycetes, while laboratory gardens only harbored *C. globosum*, which did not occur in the field.

**FIGURE 5:**
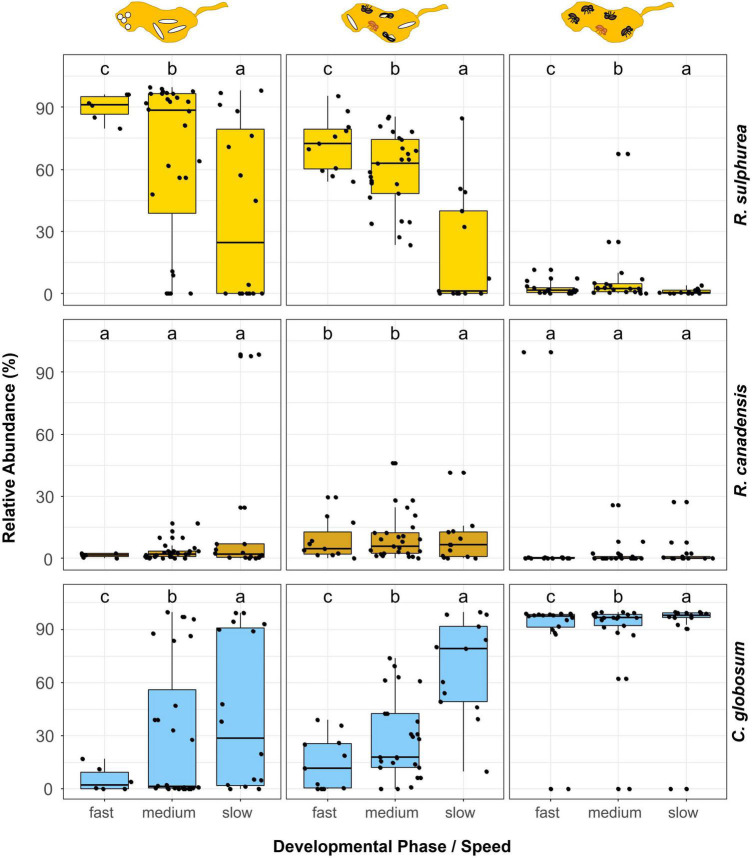
Comparison of the relative abundances of the three major fungi in laboratory fungus gardens in relation to the phases and speed of development of *X. saxesenii* nests. Box-plots with the relative abundance of the fungal mutualists *R. sulphurea* and *R. canadensis* as well as the fungal antagonist *C. globosum* for the different phases and speeds of nest development are given. Lowercase letters indicate significant differences between groups (Tukey’s HSD test: *p* < 0.05).

Changes in relative abundances of these most common taxa over the development of nests were hardly to detect, but some effects were observed. First, within field fungus gardens, the unknown species of Sordariomycetes decreased in abundance in the course of development (LMM: χ^2^ = 8.34, *p* = 0.015; EMM: “P1–P3” χ^2^ = 2.82, *p* = 0.034) (Figure 4; Supplementary Figure 12). Such a decrease of abundance was also found for the main nutritional mutualist of *X. saxesenii*, *R. sulphurea*, in laboratory nests (LMM: χ^2^ = 772.47, *p* = < 0.001; EMM: all contrasts *p* = < 0.001; Figures 4, 5). Interestingly, the two “extreme” nests that developed very quickly or very slowly showed lower abundances of this fungus (LMM: χ^2^ = 40.55, *p* = < 0.001; EMM: all contrasts *p* = < 0.001; Figure 5). The abundance of the second mutualist, *R. canadensis*, is highest in nests of early dispersing foundresses (EMM: “early–late” *p* = 0.008; “early-middle” *p* = 0.031; Supplementary Figure 13). Additionally, we found significant lower abundance of *R. canadensis* in the third phase of late dispersing females (EMM: “early–late” *p* = < 0.001; “late-middle” *p* = 0.001; Supplementary Figure 13). Finally, within laboratory fungus gardens, relative abundance of *C. globosum* increased over the course of development (LMM: χ^2^ = 13.98, *p* = 0.001) and there was a strong interaction between development phase and speed (LMM: “phase/speed” χ^2^ = 74.90, *p* = < 0.001; Figure 5). Fast developing nests had significantly less *C. globosum* present, then medium and slow developing nests both during the presence of only immatures or immature and adult offspring (phase 1 and 2) (phase 1: EMM: “medium-fast” *p* = 0.023; “fast-slow” *p* = 0.004; phase 2: “medium-slow” and “fast-slow” *p* = < 0.001; “medium-fast” *p* = 0.001). This effect disappeared when only adults were present within nests, since *C. globosum* was the most abundant taxon in almost all galleries (Figure 5).

### 3.5. Influence of bacterial and fungal communities on each other

Field samples revealed a positive linear correlation between the dissimilarity matrices of bacterial and fungal communities (*r* = 0.271; *p* = 0.002), but this correlation was not found in the laboratory samples (*r* = 0.047; *p* = 0.130).

## 4. Discussion

The analysis of the fungal symbionts in fungus gardens during *X. saxesenii* development in both field and laboratory originating nests provided us with several new insights. As expected, we detected a much higher diversity of both bacteria and fungi in the field compared to the laboratory. Fungus gardens in the laboratory are reduced to the community of bacteria and fungi that is vertically transmitted may be most essential for the beetles’ nutrition and development (Figure 3). Besides these taxa present in lab nests, field samples included additional taxa, which can be classified as plant saprophytes, endophytes, or pathogens, as well as beetle endosymbionts. While the diversity and abundance of bacterial communities of fungus gardens were relatively stable (in both lab and field) over developmental time, fungal communities shifted quite a bit. For example, especially in the laboratory nests, we observed a striking turnover from a nutritional fungal mutualist (*R. sulphurea*) to a putative lab contaminant (*C. globosum*). However, based on our mock community data (Supplementary Figure 3), we suspect a sequencing bias toward *C. globosum* here, representing a higher abundance of this species, when in fact it is slightly lower. This has already been addressed by Nuotclà et al. (2021). We observed functional beetle-symbiont interactions, specifically showing that fast development is linked with a higher abundance of *Raffaelea* fungi at least during the first phases of nest development (i.e., when immature brood was still present).

Overall bacterial communities of fungus gardens resembled taxa found in other studies, especially for ambrosia beetles associated with *Raffaelea* fungal symbionts (i.e., *Xyleborus*, *Xyleborinus*, Platypodinae; Fabig, 2011; Aylward et al., 2014; Nuotclà et al., 2021; Nones et al., 2022). These are taxa in the Alpha- Beta- and Gammaproteobacteria, as well as Actinobacteria and Bacteroidetes (Ibarra-Juarez et al., 2020; Joseph and Keyhani, 2021). Focusing only on the changes in bacterial-community composition in the field, we found a decrease of Sphingobacteriia and Flavobacteriia (Bacteroidetes) over development phases; Sphingobacteriia can exhibit xylanolytic activity (Zhou et al., 2009). Actinobacteria in return, happened to be more abundant in some galleries with only adult individuals present; they are known for their antimicrobial metabolites (Van Arnam et al., 2018; Grubbs et al., 2020). It is unclear if they are absent in the laboratory. By comparing field with laboratory nests, we found a shift from relatively heterogeneous and balanced bacterial communities to ones dominated by Gammaproteobacteria. Specifically, a *Pseudoxanthomonas* sp. showed a mean relative abundance above 80% in all three development phases. The exact role of this specific bacterium in the context of bark beetles is still unknown, but it can be often found within the communities (Nuotclà et al., 2021; Nones et al., 2022). Another Gammaproteobacterium, *Erwinia*, was also both present in the field and laboratory; it might contribute to nitrogen fixation in the system (Papen and Werner, 1979). Future work needs to address potential functional roles of bacteria in the fungus gardens of *X. saxesenii* and this study points out the few taxa present in both lab and field that should be considered first.

While bacterial communities were relatively stable, both field and laboratory galleries showed strong shifts of fungal communities in the course of nest development. This change was manifested mostly in abundances but not diversities. In the field, the strongest shifts of abundances were observed for an unknown Sordariomycetes, which decreased with nest development, and a *Graphium* sp., which increased (Figures 3, 4). Both taxa are known as symbionts of some ambrosia beetles (Harrington, 2005; Kolaøík et al., 2015), but for *X. saxesenii* both species are probably not essential, because they were missing in laboratory nests. By contrast only the two *Raffaelea* nutritional mutualists occurred both in the field and in the laboratory. Changes in abundances in relation to nest development were relatively equal between lab and field, showing both a relatively stable abundance of *R. canadensis* and a decrease of *R. sulphurea* over development phases (Figures 4, 5). This corresponds with the preference of *R. sulphurea* for moister conditions (i.e., substrates dry out over time) and its function as larval food (Nuotclà et al., 2021). It is quite likely that these fungi jointly complement the diet of the beetles also as they can co-occur on agar plates with no sign of inhibition (Biedermann, unpubl. data). A similar co-occurrence of two mutualists has been observed for the bark beetle *Dendroctonus ponderosae* (Six and Bentz, 2007).

Apart from *C. globosum*, which may be a laboratory contaminant because it occurred only in laboratory nests, fungus garden symbionts in laboratory nests were reduced to the community of bacteria and fungi that are necessary for beetle nutrition. This bottleneck effect can inform us about the functional relevance of certain bacterial and fungal taxa for beetle fitness and shows that the majority of microbial associates in the field are possibly hitchhikers on beetles’ surfaces (Birkemoe et al., 2018; Seibold et al., 2019). Metabarcoding studies of symbiont communities of ambrosia beetles in the field (e.g., Kostovcik et al., 2015; Malacrinò et al., 2017; Rassati et al., 2019) have revealed hundreds of potential beetle associates, but our laboratory findings indicate that the majority of them are not necessarily needed by the beetles. The combination of culture-dependent studies and independent methods allow for a comprehensive approach and can highlight the intensity of the associations (e.g., Kostovcik et al., 2015). Also in the context of the community detected by our method, we agree with this opinion and confirmed already isolated associates from previous studies (Batra, 1966; Francke-Grosmann, 1975; Biedermann et al., 2013). Nevertheless, as the laboratory conditions cannot mirror all possible abiotic and biotic scenarios that the beetles may face in nature, for example due to a lacking of both plant secondary compounds and less recalcitrant plant polymers in the artificial substrate (Biedermann et al., 2009), it is possible that some microbial symbionts (mutualistic, commensalistic, or pathogenic) got lost under laboratory conditions, while actually playing important roles in nature. Finally, more homogenous temperature and humidity conditions in the laboratory may have also led to the competitive exclusion of some taxa (e.g., Hibbing et al., 2010).

Using our laboratory nests, we could show that fungal communities had a strong influence on the speed of nest development (Supplementary Figures 4, 5). There is a clear succession from a *R. sulphurea* dominated first nest phase (with immature brood) to a mixed *R. sulphurea*, *R. canadensis* and *C. globosum* community in the second nest phase (with immature and adult brood) and a last phase dominated by *C. globosum*. Interestingly, the earlier this succession moves away from *R. sulphurea*, the slower the beetle development. Fast developing nests were characterized by higher fungal richness (Supplementary Figures 4, 5), lower relative abundance of the antagonistic fungus *C. globosum* and a higher abundance of the nutritional *Raffaelea* species, in particular *R. sulphurea* (Figure 5). This is certainly an effect of the better food supply for the developing offspring, which is further corroborated by the observation that there is no correlation between speed of development and relative abundance of nutritional fungi in the third nest phase (i.e., with only adults present that finished development).

This study is the first to provide evidence for a microbe-mediated transgenerational effect of female dispersal time on development of the subsequent generation in *X. saxesenii*. Interestingly, effects of dispersal were not significant in the first phase of development, but appeared only in the second phase (with immature and adult brood). In particular, the earlier a female dispersed, the lower was the fungal diversity during the second phase in their newly established fungus gardens. The same effect was also found for the relative abundance of *R. canadensis* in the second and third phase (Supplementary Figure 13). This suggests that an early dispersal of females from their maternal nests benefits their fitness due to a higher abundance of the second food fungus, *R. canadensis* (which is most abundant during the second phase with first adult offspring present) relative to other non-beneficial fungi. As found by Nuotclà et al. (2021), *R. canadensis* enables long-lasting nests and increases offspring numbers. Therefore, delayed dispersal, which is found in many adult daughters of *X. saxesenii* that remain and socially care for brood and fungus (Peer and Taborsky, 2007; Biedermann and Taborsky, 2011), may come with a transgenerational cost of less beneficial symbiont communities later on. This can be easily explained by the above-mentioned succession from nutritional to non-beneficial fungi inside nests and the fact that relatively more non-beneficial fungi are unintentionally transmitted by the later dispersing beetles on their cuticle (Francke-Grosmann, 1975; Mayers et al., 2022).

Bacterial and fungal communities in field fungus gardens had a mutual influence on each other, while laboratory gardens did not exhibit this relationship. We posit that this difference is due to the greater diversity of bacteria in the field, which enabled more flexibility to adapt to the changing conditions caused by the developing fungal community. In contrast, the laboratory conditions represent a more stable, closed environment which is based on a sterile, semi-natural medium that limits the diversity of the microbial community, resulting in the dominance of a few core taxa and few changes over time. This finding suggests that in natural environments, the overall bacterial community within ambrosia beetle nests may be shaped by the dominant fungal species. By changing the environment with their enzymatic activity, fungi could influence, positively or negatively, the bacterial symbionts as the study by Zhang et al. (2022) reported in the context of composting. Here, the bacterial genera, *Flavobacterium* and *Pseudomonas* showed a positive correlation to the fungal genus *Aspergillus*, but a negative one with *Myceliophthora*.

Our study shows that the fungus gardens of ambrosia beetles, at least the ones from *X. saxesenii*, are very different from the ones of farming ants and termites. While the latter live in long-lived eusocial societies that maintain and stabilize growth conditions for their fungal mutualists by progressive provisioning of substrate, dead-wood substrate is not replenished by the beetles and deteriorates relatively quickly (Biedermann and Vega, 2020). Hence, we find a single cultivar dominating typical fungus gardens of ants and termites, while more and more studies in cooperatively breeding ambrosia beetles with long overlaps between immature and adult offspring (many species in the Xyleborini genera *Xyleborus* and *Xyleborinus*, possibly also Platypodinae) show that there is often a succession of different fungi (or yeasts) and at least two, possibly even more, can serve as food sources (this study; Ibarra-Juarez et al., 2020; Diehl et al., 2022; Menocal et al., 2023). Alternatively, other less social ambrosia beetles have relatively short-lived nests (only one generation per nest, typically little overlap between immature and adult offspring) and rely on only single fungal cultivars [e.g., the Xyloterini and the Xyleborini genera *Xylosandrus* and *Anisandrus*; (Kostovcik et al., 2015; Mayers et al., 2015, 2020)]. Overall, ambrosia beetles are unable to stabilize the community, such as other ants or termite farmers do, because they cannot replenish the fungal substrate. The only exception might be the few Platypodinae ambrosia beetles that colonize living trees (without killing them; Kirkendall et al., 2015), in which trees may replenish the nutrients used by the growing fungi. Communities of their fungus gardens have not been studied, so far. What also remains unclear is if and how multiple cultivars in ambrosia beetle nests respond to changing temperatures, moisture and switches of tree hosts (most of these ambrosia beetle species are tree-host generalists).

The first direct comparison of microbial communities in ambrosia beetle fungus gardens of field and laboratory nests revealed a strong reduction of both bacterial and fungal associations in laboratory nests. We argue that these few taxa are essential mutualists, needed for *X. saxesenii* reproduction and development; more complex substrate in real wood may require additional taxa, however. Furthermore, we observed in both, field and laboratory, a succession of fungal symbionts during the course of beetle development, in which nutritional fungal mutualists are slowly replenished by non-beneficial fungi. The quicker the succession proceeds, the slower nests are developing, which certainly relates to the diminishing food supply. Finally, this fungal succession might also have transgenerational costs for delayed dispersing daughters, as early dispersing daughters transmit the more beneficial fungal communities for founding new fungus gardens for their future offspring. Future studies should focus now on revealing the functional roles of the putatively beneficial bacterial taxa that were present in both field and laboratory nests. Furthermore, we strongly recommend to include environmental controls (i.e., non-colonized wood and media next to nests), which we missed and therefore we could not safely discern vertically transmitted from environmentally acquired symbionts.

## Data availability statement

The datasets presented in this study can be found in online repositories. The names of the repository/repositories and accession number(s) can be found below: https://github.com/janinad88/microbial-succession-of-ambrosia-beetle-galleries, NA; https://www.ncbi.nlm.nih.gov/, PRJNA915190.

## Author contributions

JD and PB conceived and designed the experiments. JD carried out the study and analyzed the data. AK helped in the bioinformatic and statistical processing. JD, AK, and PB wrote the manuscript. All authors contributed to the article and approved the submitted version.
