# Supplementary material for: Comparing the succession of microbial communities throughout development in field and laboratory nests of the ambrosia beetle Xyleborinus saxesenii
Source: Front Microbiol. 2023 Apr 20;14:1151208. doi: 10.3389/fmicb.2023.1151208 (PMC10159272; doi:10.3389/fmicb.2023.1151208)
Supplement: Supplementary file 1 [file Data_Sheet_1.DOCX]

Supplementary Material

To publication **Comparing the succession of microbial communities throughout development in field and laboratory nests of the ambrosia beetle *Xyleborinus saxesenii*** by **JMC Diehl, A Keller & PHW Biedermann** in Front. Microbiol.

GitHub Repository: https://github.com/janinad88/microbial-succession-of-ambrosia-beetle-galleries

Raw Sequence Data available on NCBI SRA under BioProjectID: PRJNA915190

**Supplementary Methods**

After running the contaminant removal method with the package ‘decontam’ (Davis et al., 2018), ‘negative’ control samples (*N_field_* = 4; *N_lab_* = 14) identified 163 of the bacterial 16S ribosomal RNA and 3 of the fungal 28S ribosomal RNA ASVs as external contaminants from the field samples. Whereas, 430 in the laboratory nests of the bacterial 16S ribosomal RNA and 16 of the fungal 28S ribosomal RNA ASVs were found. Overall the quality of field samples was better and showed less contaminations. The filtering process for ‘decontam’ reduces the complexity of microbiome data while preserving its integrity in downstream analysis. By reducting the classification methods' sensitivity and technical variability, it allows researchers to generate more reproducible and better comparable results in microbiome data analysis (Cao et al., 2021).

Species accumulation curves (Supplementary Figure 3) showed that most samples were sequenced to saturation after approximately 20,000 high quality reads for 16S and 28S. The insufficient sequencing effort to represent the entire microbial communities in some of the samples could be due to a high amount of single read copies. Taxa that could not be assigned further than to Kingdom/Domain level, were removed prior to follow-up analyses.


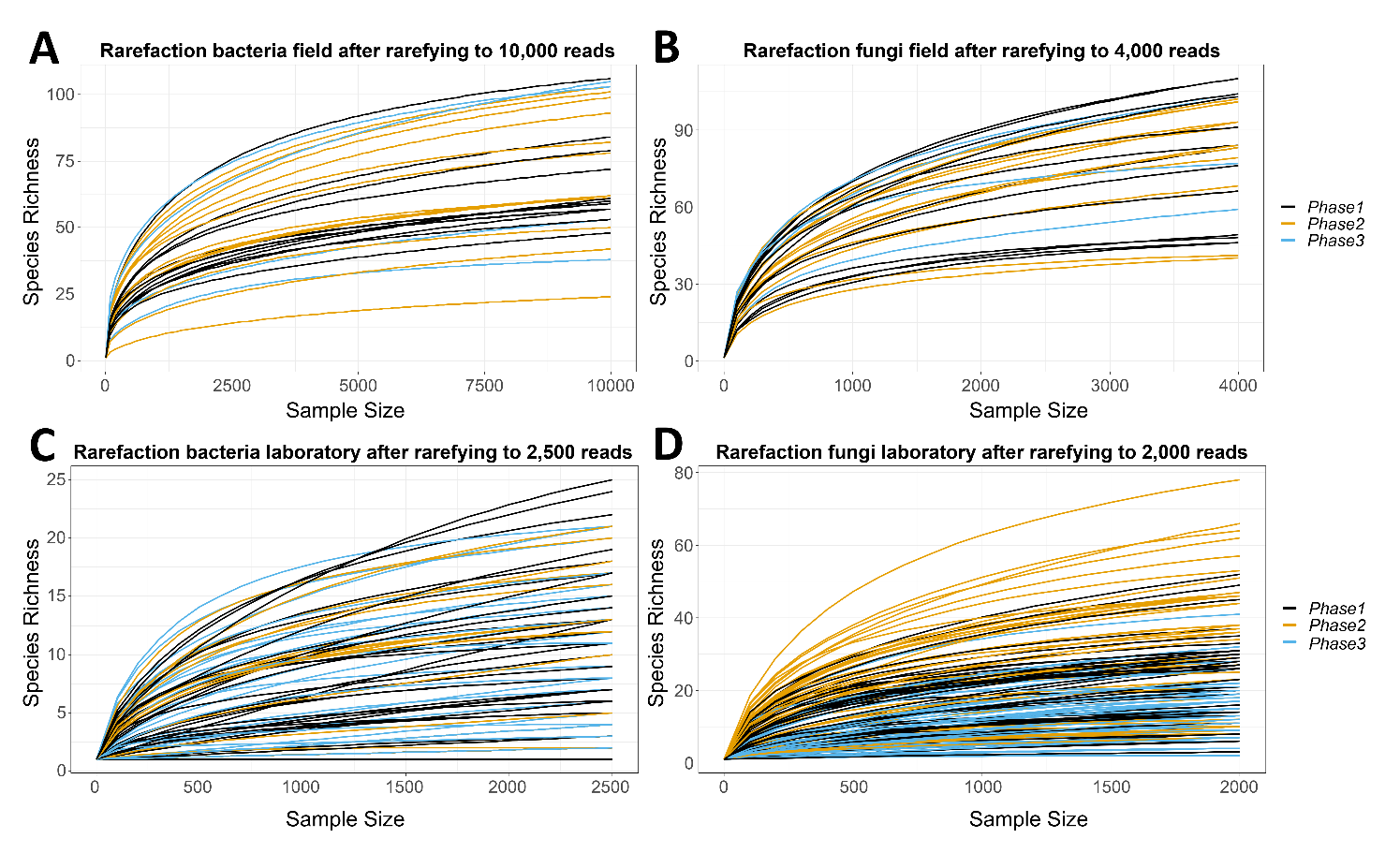


Supplementary Figure 1. Rarefaction curves of amplicon sequence variants in the datasets after running 'decontam' filtering step and excluding sequence control samples separated by ‘*developmental phases*’. (A) Samples of field nests from *X. saxesenii* in beech logs sequenced with 16S primers to detect the bacterial community. Dataset rarefied to 10,000 reads for the analysis of alpha diversity analysis. (B) Same samples of field nests sequenced with 28S primers to detect the fungal community. Dataset rarefied to 4,000 reads for the analysis of alpha diversity analysis. (C) Samples of laboratory nests from *X. saxesenii* in artificial beech rearing medium sequenced with 16S primers to detect the bacterial community. Dataset rarefied to 2,500 reads for the analysis of alpha diversity analysis. (D) Same samples of laboratory nests sequenced with 28S primers to detect the fungal community. Dataset rarefied to 2,000 reads for the analysis of alpha diversity analysis.

Supplementary Table 1. Number of replicates used for analysis in the different sequencing datasets.

| Dataset | Replicates  Phase 1 | Replicates  Phase 2 | Replicates  Phase 3 |
| --- | --- | --- | --- |
| 16S field | 13 | 13 | 4 |
| 28S field | 13 | 13 | 4 |
| 16S laboratory | 32 | 27 | 30 |
| 28S laboratory | 50 | 49 | 49 |


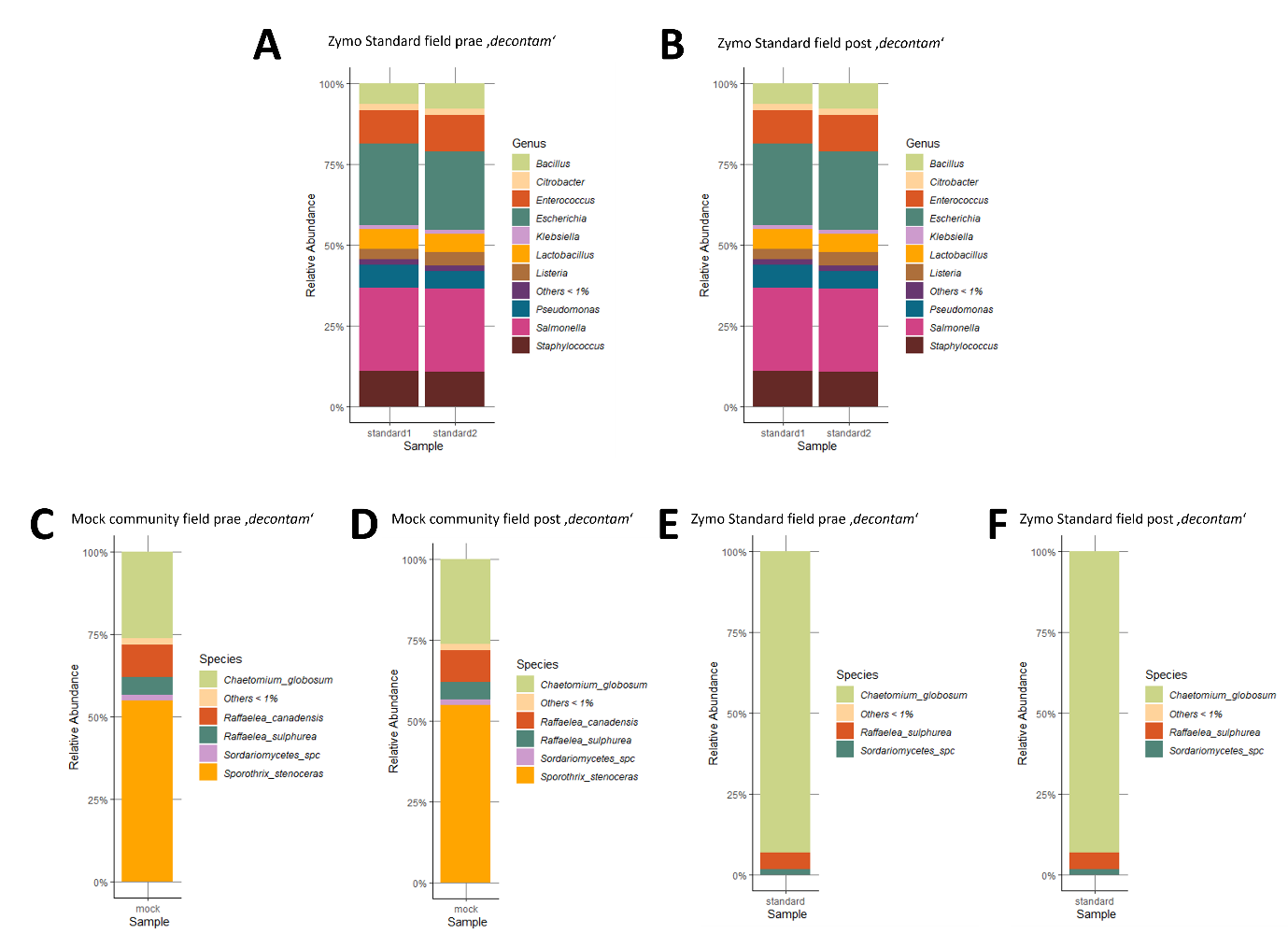


Supplementary Figure 2. Relative Abundance of detected taxa in the sequencing control samples of the field samples prae und post filtering with the ‘decontam’ package. (A) ZymoBIOMICS™ Microbial Community Standard prae removal of bacterial contaminants (standard 1 = 12,661 reads; standard 2 = 14,118 reads). (B) ZymoBIOMICS™ Microbial Community Standard post removal of bacterial contaminants (standard 1 = 12,661 reads; standard 2 = 14,118 reads). The Zymo Microbial Community Standard should contain the following eight bacterial genera: *Pseudomonas, Escherichia, Salmonella, Lactobacillus, Enterococcus, Staphylococcus, Listeria, Bacillus* (ZymoBIOMICS™ Microbial Community Standard Instruction Manual). These could all be detected in our sequencing. (C) Mock community prae removal of fungal contaminants. Equal amounts of biomass were used to create the community. (32,739 reads) (D) Mock community post removal of fungal contaminants (32,674 reads). (E) ZymoBIOMICS™ Microbial Community Standard prae removal of fungal contaminants (59 reads). (F) ZymoBIOMICS™ Microbial Community Standard post removal of fungal contaminants (59 reads). These Mock communities should contain two fungal genera: *Cryptococcus* and *Saccharomyces* (Instruction Manual) which both could not be amplified with our primers. Instead, the Ambrosia beetle fungi appear, however the extremely low read numbers in these samples demonstrate that only neglectable cross contamination was sequenced.


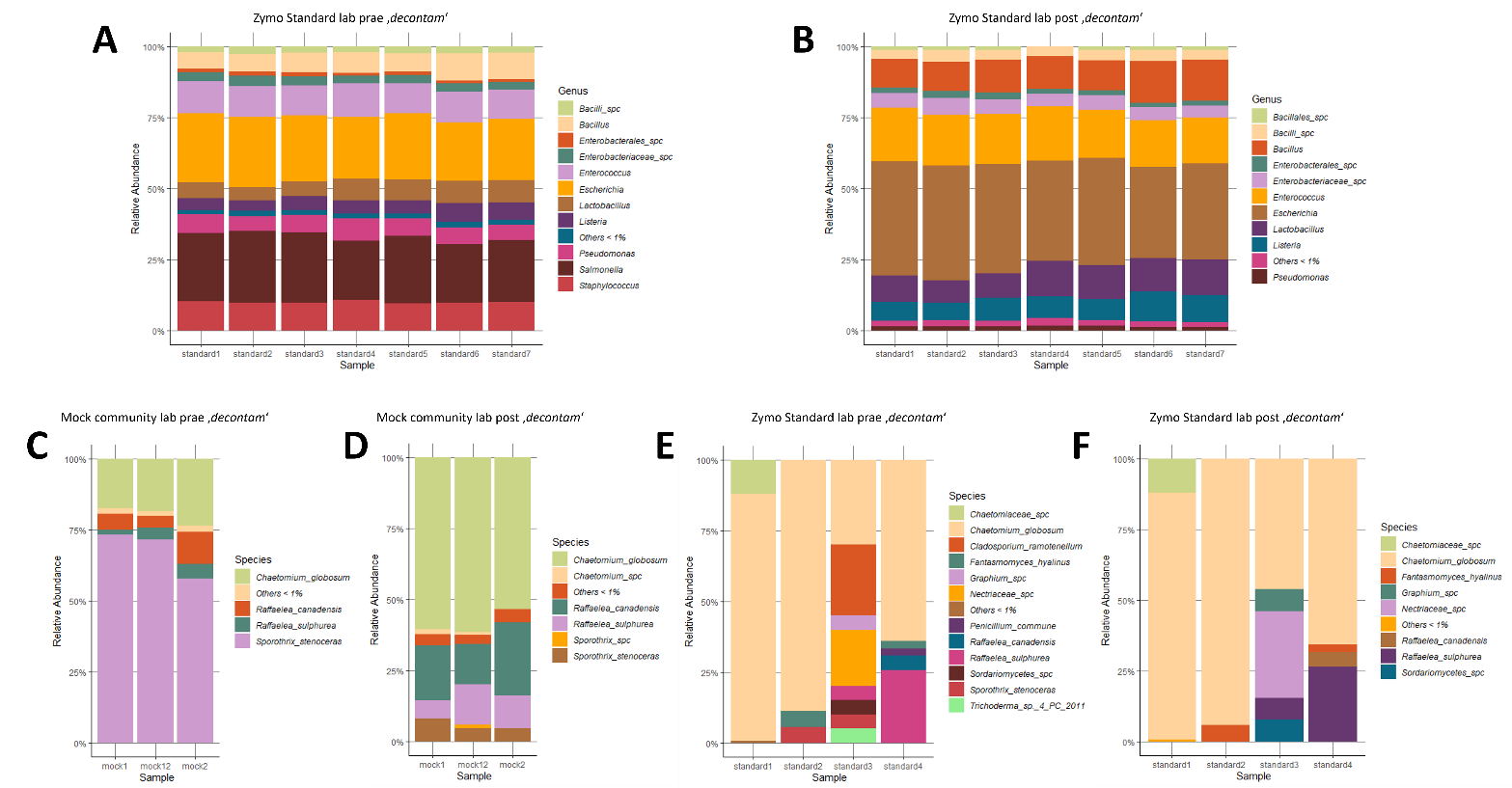


Supplementary Figure 3. Relative Abundance of detected taxa in the sequencing control samples of the laboratory samples prae und post filtering with the ‘decontam’ package. (A) ZymoBIOMICS™ Microbial Community Standard prae removal of bacterial contaminants (average 22,405 reads). (B) ZymoBIOMICS™ Microbial Community Standard post removal of bacterial contaminants (average 13,804 reads). The Zymo Microbial Community Standard should contain the following eight bacterial genera: *Pseudomonas, Escherichia, Salmonella, Lactobacillus, Enterococcus, Staphylococcus, Listeria, Bacillus* (ZymoBIOMICS™ Microbial Community Standard Instruction Manual). These could all be detected in our sequencing, however, the decontamination step removed *Staphylococcus* as this also appeared as a contaminant in the negative controls and could not be found in our true samples. (C) Mock community prae removal of contaminants. Equal amounts of biomass were used to create the community. (average 23,271 reads) (D) Mock community post removal of contaminants (average 7,638 reads). (E) ZymoBIOMICS™ Microbial Community Standard prae removal of fungal contaminants (average 627 reads). (F) ZymoBIOMICS™ Microbial Community Standard post removal of fungal contaminants (average 625 reads). These Mock communities should contain two fungal genera: *Cryptococcus* and *Saccharomyces* (Instruction Manual) which both could not be amplified with our primers. Instead, the Ambrosia beetle fungi appear, however the extremely low read numbers in these samples demonstrate that only neglectable cross contamination was sequenced.

**Supplementary Results**

**Sequencing controls**

Controls showed a sufficient sequencing result of the microbial community standard from ZymboBiomics. All bacterial species contained in the standard are represented in the bar graph of relative bacterial taxa abundance (Supplementary Figure 1A & Supplementary Figure 2A) and appeared rarely in the other samples (≤105 reads/sample, only for *Staphylococcus aureus*). Negative controls (autoclaved rearing medium for beetle breeding and PCR water control) showed some bacterial species which can be neglected, since the first control of these samples with gel electrophoresis ahead to sequencing revealed no visible bands and rarefaction curves and richness estimates suggest a low input of single sequences due to possible cross-contamination. Due to a very low read number of these ASVs in our samples (≤ 480 reads/sample, *Streptomyces sp.*) we choose the low abundance filtering method. Similarly, a closer look on the fungal controls yielded important information on the quality of our 28S MiSeq primers. Our own mock community of known fungi associated with *X. saxesenni* (*R. sulphurea, R. canadensis, C. globosum, Ophiostoma stenosterans* and *Pichia sp.*) revealed like in Nuotcla et al. (2021) and Diehl et al. (2022) that the two symbionts *R. canadensis* and *R. sulphurea* can be distinguished, as well as other fungi of the orders Eurotiales, Sordariales, Hypocreales, Capnodiales, Onygenales and Dothideales, but again the yeasts including Saccharomycetales (e.g. *Pichia sp., Candida sp.*) were not differentiated (Supplementary Figure 1C & Supplementary Figure 2C). The negative controls showed only few reads (≤ 418 reads, *C. globosum*) of fungal taxa. We again decided to neglect this information since all negative controls (*N* = 6) revealed in gel electrophoresis ahead to sequencing no visible bands and rarefaction curves as well as richness estimates again suggest a low input of single sequences due to potential cross-contamination.

**Bacterial beta diversity**

Beta diversity slightly differed between phase 1, 2 and 3 (PERMANOVA_Field_: phase1 vs. phase2: *R^2^* = 0.078, *F* = 2.43, *p* = 0.014; phase1 vs. phase3: *R^2^* = 0.115, *F* = 2.36, *p* = 0.010), but not between phase 2 and 3 (*R^2^* = 0.039, *F* = 0.714, *p* = 0.720) in the field. The NMDS plot of Bray-Curtis dissimilarity displayed some separation of the phases within the sampled trees (Supplementary Figure 6). The homogeneity groups between the developmental variable demonstrated an equal compositional variance (Betadisper: *F* = 1.64, *p* = 0.213), whereas the groups of the originating trees did not (Betadisper: *F* = 17.30, *p* = <0.001).

In laboratory nests pairwise comparison on the basis of PERMANOVA including the phase and development speed showed that dispersion of the developmental speed differed between the first and third phase (*R^2^* = 0.061, *F* = 1.89, *p* = 0.024). No statistically different dispersion was found between the first and second (‘phase’ *p* = 0.359, ‘speed’ *p* = 0.936) or second and third phase (‘phase’ *p* = 0.335, ‘development’ *p* = 0.843). The NMDS plot of Bray-Curtis dissimilarity displayed no separation of the phases or development speed (Supplementary Figure 9). The homogeneity groups between the developmental variables and dispersal time demonstrated an equal compositional variance (Betadisper: ‘phase’ *F* = 0.867, *p* = 0.424, ‘speed’ *F* = 1.46, *p* = 0.238, ‘dispersal’ *F* = 2.10, *p* = 0.129).

**Fungal beta diversity**

Adjusted p-values of pairwise PERMANOVA demonstrated significant variation between the first and third phase (*R^2^* = 0.237, *F* = 5.26, *p* = 0.001) and between the first and second (*R^2^* = 0.079, *F* = 2.24, *p* = 0.030) in the field. No separation was found for the second and third developmental phases (*R^2^* = 0.080, *F* = 1.47, *p* = 0.167). Instead, looking at these two groups, the pairwise PERMANOVA showed a marginal significant effect of the tree nests originated from (*R^2^* = 0.261, *F* = 1.59, *p* = 0.083), as well as in the first and second phase comparison (*R^2^* = 0.147, *F* = 2.08, *p* = 0.091). In the NMDS plot of Bray-Curtis dissimilarity some variation of the phases, where the dispersion of samples in the groups gets bigger the older the fungus garden, is displayed. (Supplementary Figure 7). This heterogeneity of groups between the developmental phase demonstrates more compositional variance and is reflected by the significant test of homogeneity (Betadisper: *F* = 4.17, *p* = 0.026). As in the bacterial composition, we found this effect for the originating tree for the samples, too (Betadisper: *F* = 3.82, *p* = 0.022).

In lab fungus gardens, adjusted p-values of pairwise PERMANOVA demonstrated significant variation between the first and third phase (*R^2^* = 0.391, *F* = 63.51, *p* = 0.001) with no influence of the speed (*R^2^* = 0.025, *F* = 2.03, *p* = 0.246), and between the second and third (‘phase’ *R^2^* = 0.443, *F* = 87.68, *p* = 0.001, ‘speed’ *R^2^* = 0.083, *F* = 8.19, *p* = 0.006) (Supplementary Figure 10). No significant separation was found for the first and second developmental phases (‘phase’ *R^2^* = 0.022, *F* = 2.71, *p* = 0.194, ‘speed’ *R^2^* = 0.196, *F* = 11.90, *p* = 0.071). In the NMDS plot of Bray-Curtis dissimilarity some variation of the phases, where the dispersion of samples in the groups gets bigger for the second phase and a shift from first to third phase is displayed. (Supplementary Figure 10). This heterogeneity of groups between the developmental phase and speed demonstrates more compositional variance and is reflected by the significant test of homogeneity (Betadisper: ‘phase’ *F* = 18.91, *p* = <0.001, ‘speed’ *F* = 2.50, *p* = 0.086).

**Supplementary Figures and Tables**


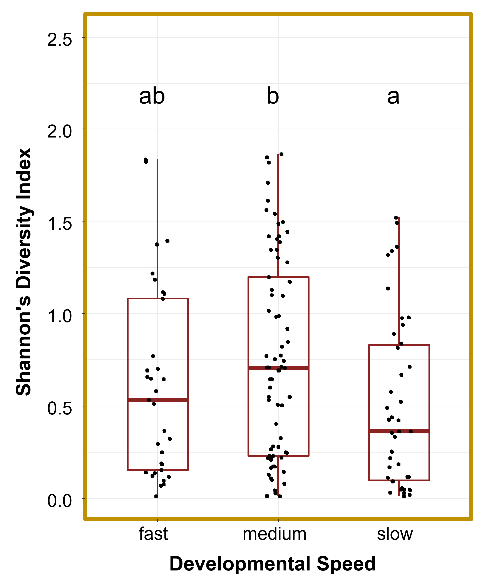


Supplementary Figure 4. Boxplots of Shannon’s diversity indices in the three developmental speeds for the fungal communities in laboratory nests. Here, ‘medium’ developing nests had a higher Shannon’s diversity index than ‘slow’ developing ones. Lowercase letters indicate significant differences between groups (*p* < 0.05; Tukey's test).


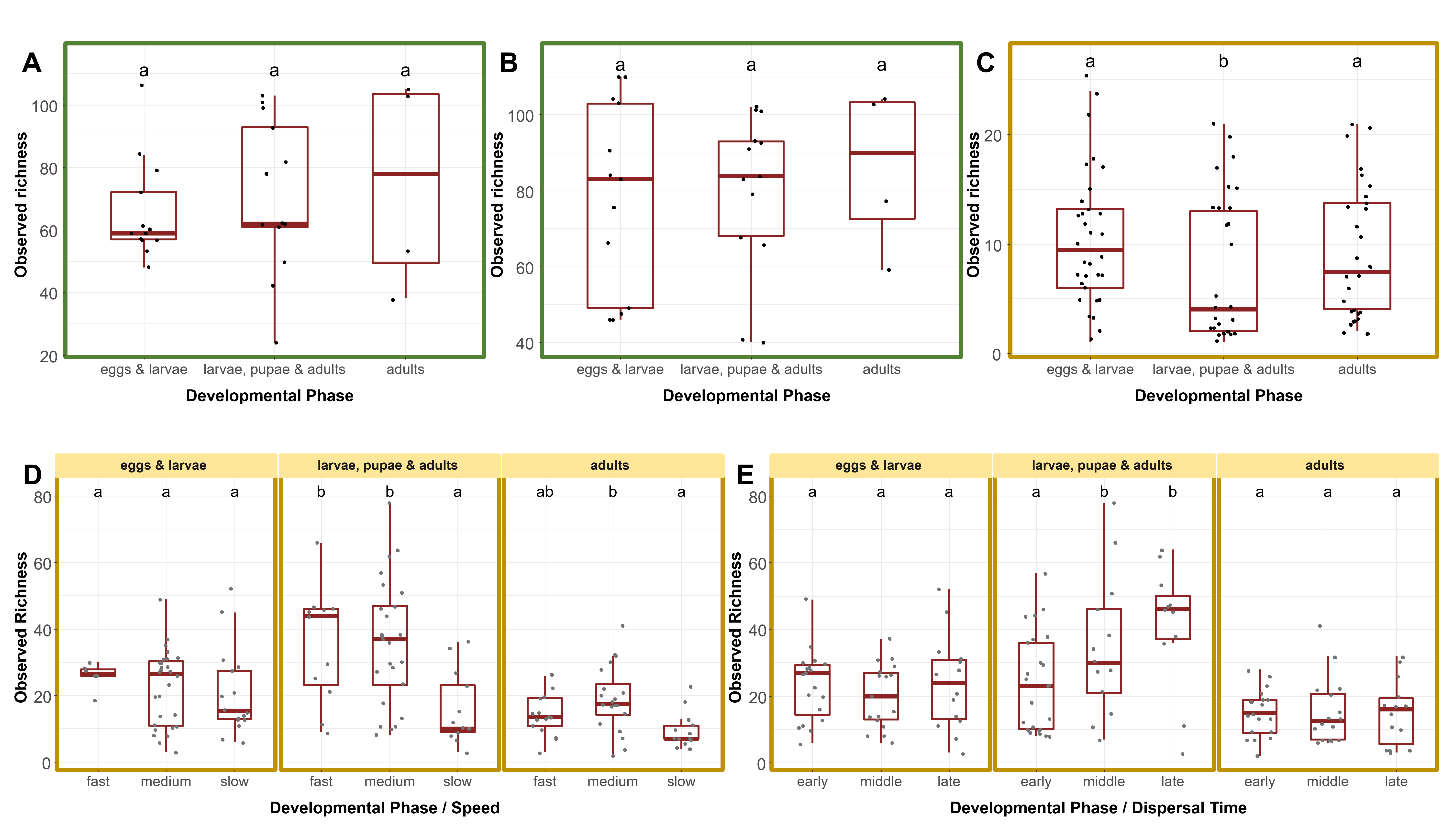


Supplementary Figure 5. Boxplots of observed richness estimates in the three developmental phases for the bacterial (A+C) and fungal (B, D & E) communities in field (green) and laboratory (yellow) nests. Field nests showed no significant differences between the developmental phases in both bacterial (A) and fungal (B) observed richness. Laboratory nests, on the other hand, had lower observed richness in the bacterial communities in the phase with larvae, pupae and adults present compared to only eggs and larvae or only adults (C). The fungal communities in laboratory nests, moreover, pointed out interactions where richness was as well influenced by the developmental speed (D), as well as the dispersal time of foundresses (E). In both interaction differences could be found in the second phase with larvae, pupae and adults present. Here, ‘fast’ and ‘medium’ developing nests or nests from ‘middle’ and ‘late’ dispersing foundresses had a higher fungal richness. Lowercase letters indicate significant differences between groups (*p* < 0.05; Tukey's test).


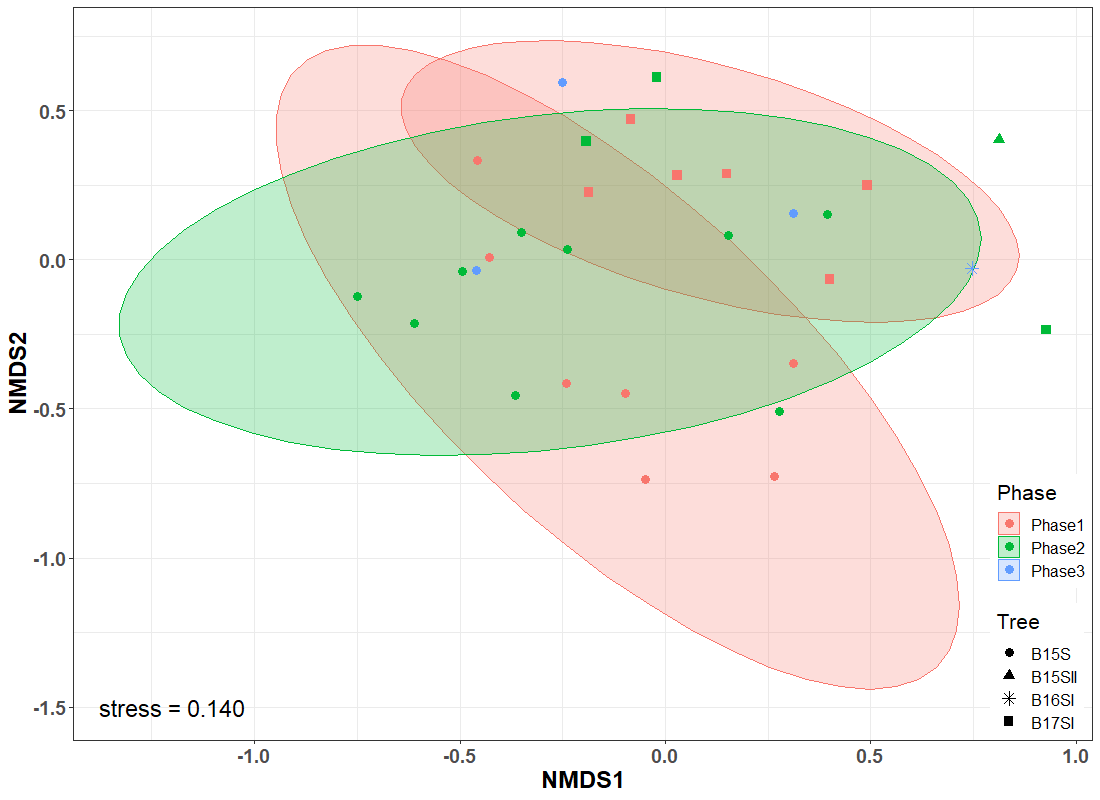


Supplementary Figure 6. The NMDS plot of Bray-Curtis dissimilarity in bacterial communities of field nests displayed some separation of the phases within the sampled tree logs.


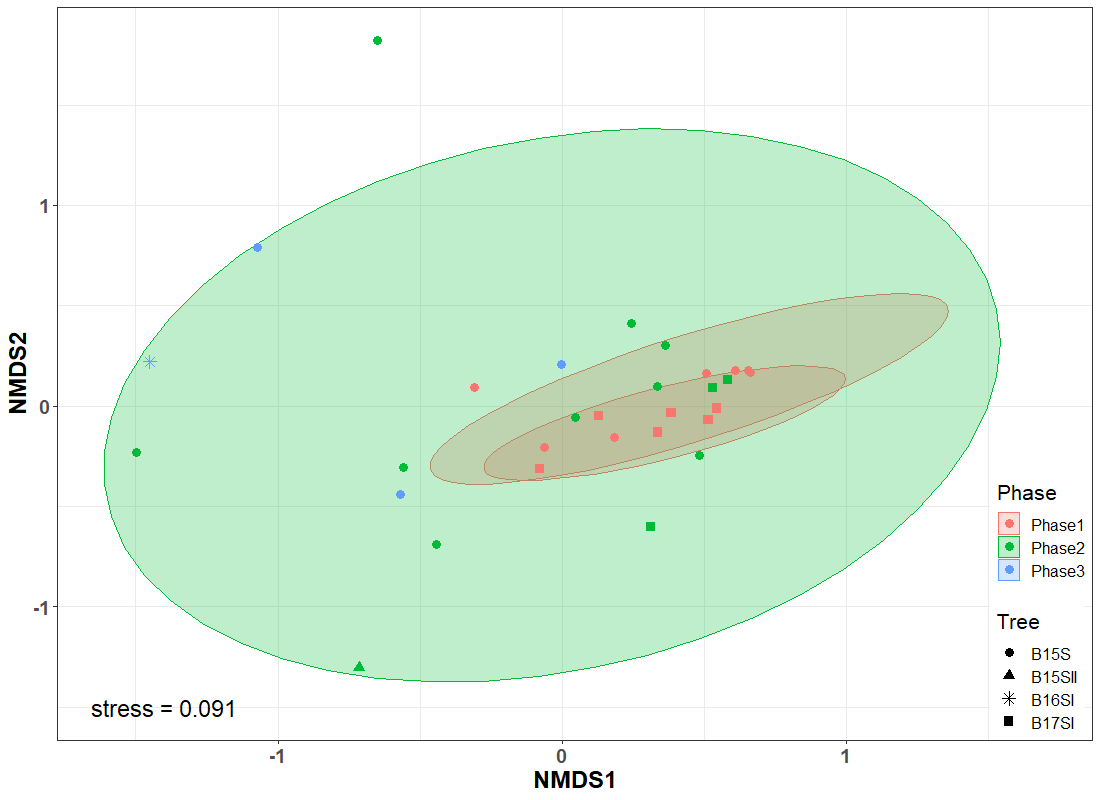


Supplementary Figure 7. The NMDS plot of Bray-Curtis dissimilarity in fungal communities of field nests displayed some variation of the phases, where dispersion of samples in the groups gets bigger the older the fungus garden.


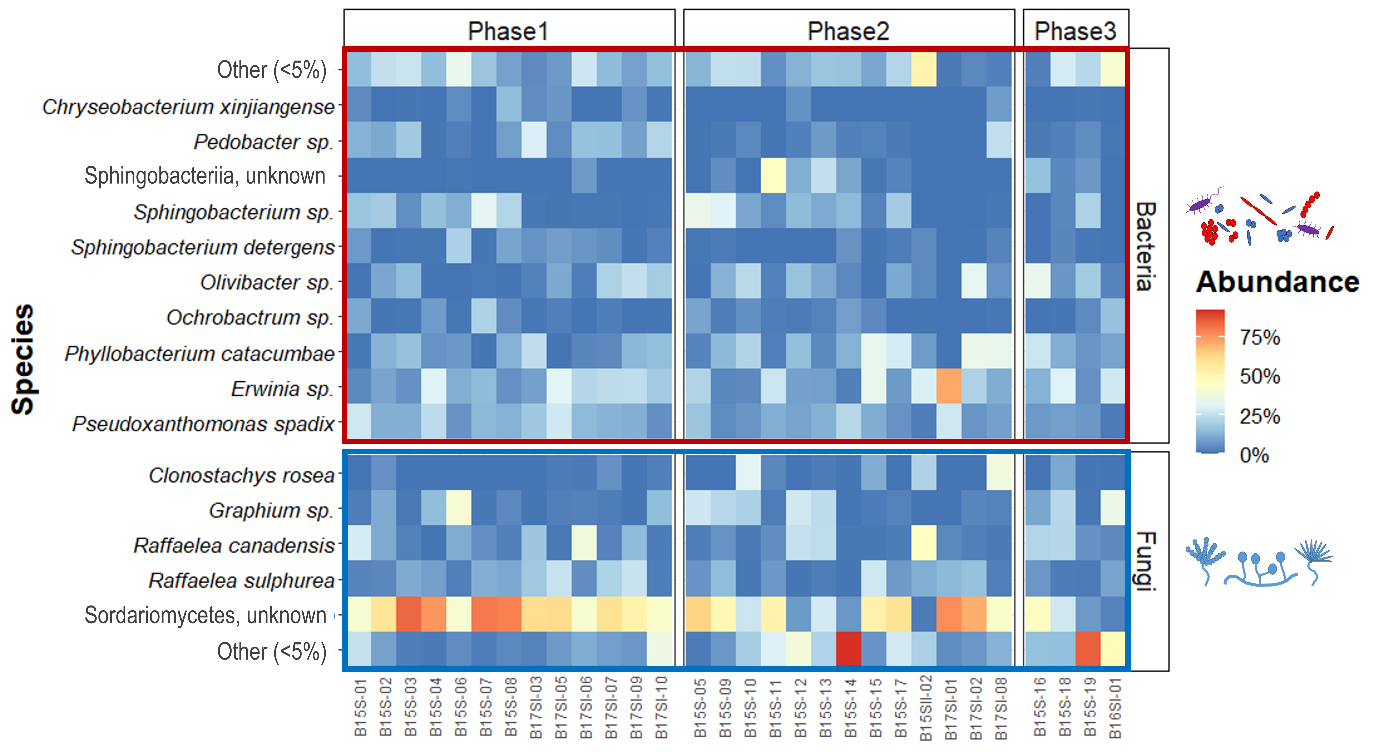


Supplementary Figure 8. Heatmap of most abundant taxa in the three developmental phases for both bacterial and fungal communities in field nests. Taxa under detection threshold of 5% relative abundance are combined into “Other”.


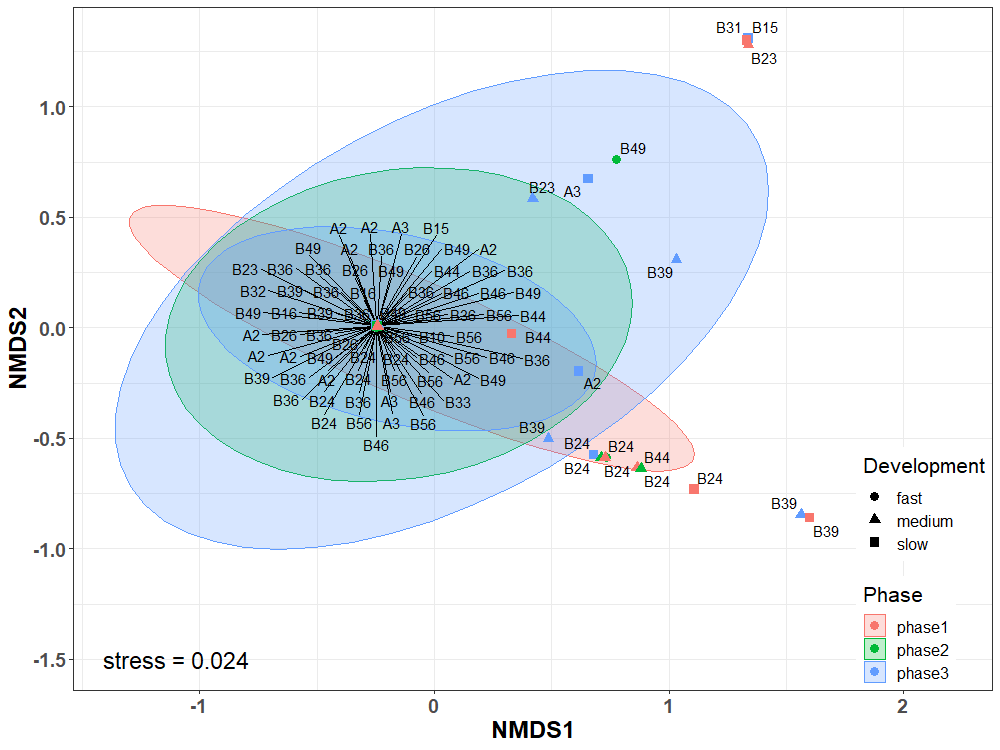


Supplementary Figure 9. The NMDS plot of Bray-Curtis dissimilarity displayed no separation of the phases or development speed in bacterial communities of laboratory nests.

Supplementary Figure 10. NMDS of Bray-Curtis dissimilarity displayed separation by phases and development speed in fungal communities of laboratory nests.


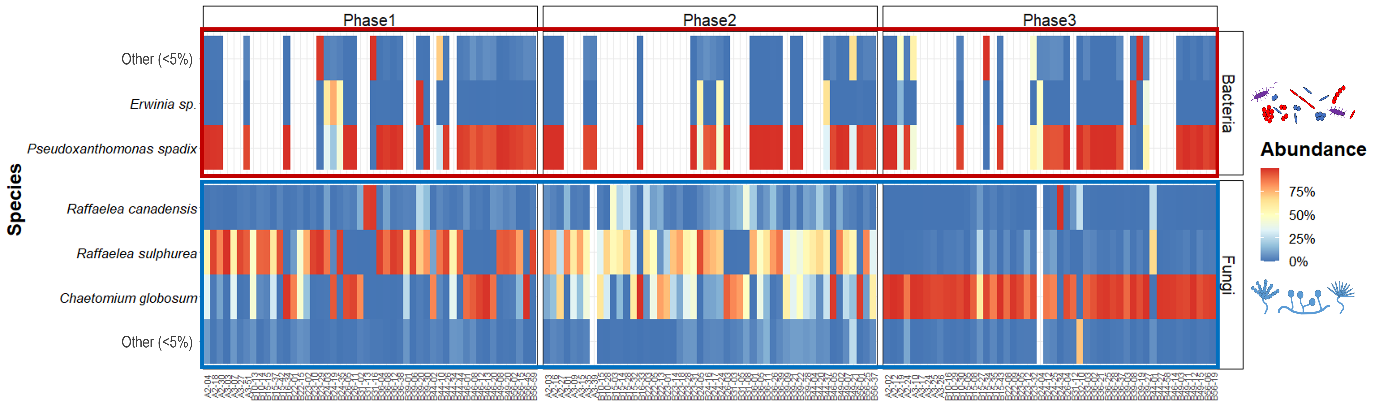


Supplementary Figure 11. Heatmap of most abundant taxa in the three developmental phases for both bacterial and fungal communities in laboratory nests.


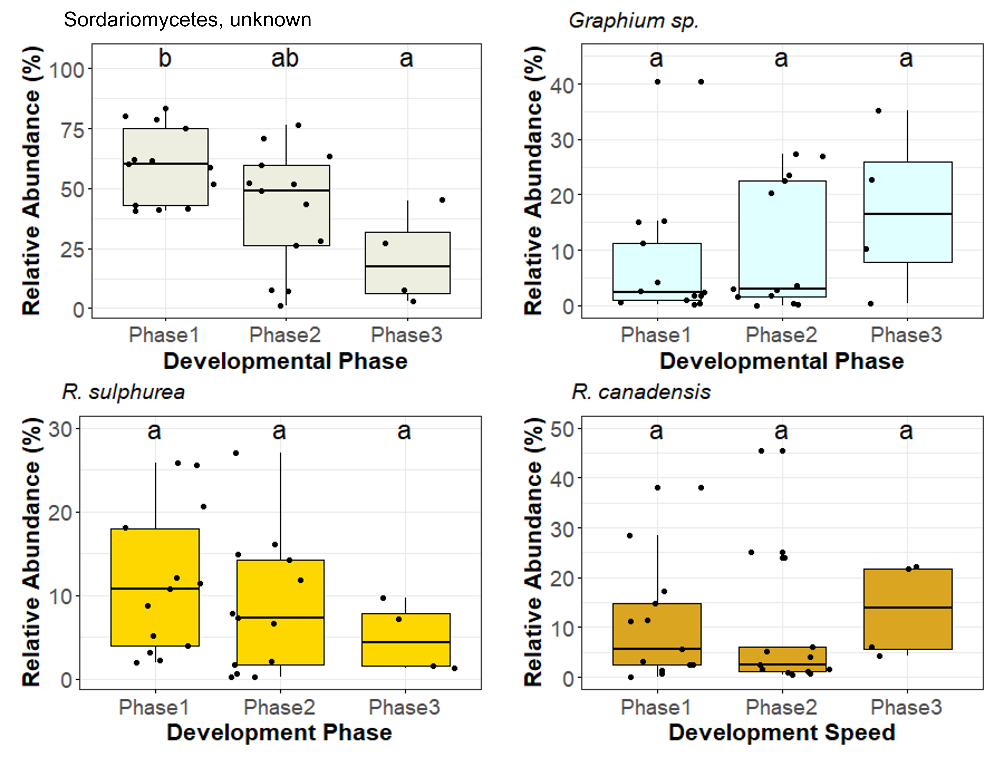


Supplementary Figure 12. Effects of the relative abundance of fungal core taxa in field nests. Direct comparison of the relative abundance of core taxa in field nests showed neither significant reduction of the primary food fungus, *R. sulphurea*, over nest development (bottom, left), nor increase of *R. canadensis* with developmental phases (bottom, right). *Graphium* sp. abundance seemed to have increased with nest age, but also revealed not significant difference between the phases (top, right). The abundance of the highly dominant unknown Sordariomycetes decreased between the first and third developmental phase significantly (top, left). Lowercase letters indicate significant differences between groups (*p* < 0.05; Tukey's test).


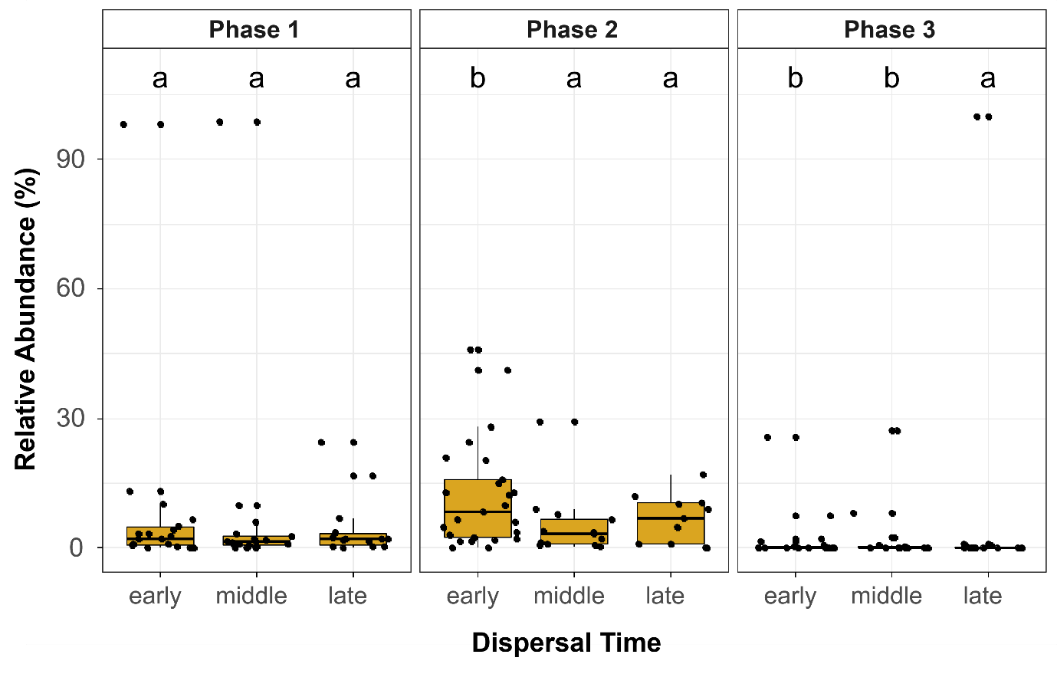


Supplementary Figure 13. Effect of the relative abundance of *R. canadensis* in laboratory nests. Direct comparison of the relative abundance of *R. canadensis* in laboratory nests showed an influence of the developmental phase with time of the foundress’s dispersal, where nests of ‘early’ dispersing foundresses in the second developmental phase had a significant higher relative abundance compared to ‘middle’ and ‘late’ dispersing ones. Lowercase letters indicate significant differences between groups (*p* < 0.05; Tukey's test).

| Fungi | | | | | | | |
| --- | --- | --- | --- | --- | --- | --- | --- |
| Order | mean | SD | N | Species | mean | SD | N |
| Xylariales | 2.56% | 6.16% | 28 | Unknown Diatrypaceae | 1.24% | 4.47% | 17 |
| Microascales | 9.96% | 11.94% | 30 | *Graphium* sp. | 9.95% | 11.94% | 29 |
| Chaetothyriales | 2.93% | 8.80% | 22 | Unknown Herpotrichiellaceae | 0.72% | 2.84% | 15 |
| Hypocreales | 11.87% | 16.72% | 28 | *Clonostachys rosea* | 2.37% | 7.05% | 35 |
|  |  |  |  | *Clonostachys* sp. | 0.81% | 1.81% | 15 |
|  |  |  |  | Unknown Hypocreales | 0.78% | 1.06% | 24 |
|  |  |  |  | *Nectria balansae* | 0.71% | 2.78% | 8 |
|  |  |  |  | *Neonectria* sp. | 1.45% | 7.18% | 13 |
|  |  |  |  | *Trichoderma deliquescens* | 2.70% | 10.99% | 12 |
| Togniniales | 1.75% | 5.20% | 18 | *Phaeoacremonium austroafrica* | 0.96% | 4.54% | 9 |
|  |  |  |  | *Phaeoacremonium* sp. | 0.79% | 2.76% | 14 |
| Ophiostomatales | 21.58% | 14.62% | 30 | *Raffaelea aff. canadensis* | 0.78% | 1.34% | 24 |
|  |  |  |  | *Raffaelea canadensis* | 10.34% | 12.13% | 30 |
|  |  |  |  | *Raffaelea sulphurea* | 9.33% | 8.0% | 30 |
|  |  |  |  | Unknown Sordariomycetes | 46.6% | 24.13% | 30 |
| Bacteria | | | | | | | |
| Class | mean | SD | N | Genus | mean | SD | N |
|  |  |  |  | Unknown Bacteriodetes | 1.42% | 6.04% | 12 |
| Flavobacteriia | 4.54% | 5.44% | 27 | *Chryseobacterium* | 3.54% | 5.09% | 26 |
|  |  |  |  | *Flavobacterium* | 0.98% | 2.55% | 15 |
| Actinobacteria | 2.83% | 4.59% | 30 | *Demetria* | 0.52% | 2.30% | 7 |
| Chitinophagia | 1.36% | 2.36% | 19 | *Taibaiella* | 1.36% | 2.36% | 19 |
| Alphaproteobacteria | 20.34% | 10.10% | 30 | *Mesorhizobium* | 0.53% | 0.72% | 22 |
|  |  |  |  | *Ochrobactrum* | 4.11% | 5.15% | 29 |
|  |  |  |  | *Phyllobacterium* | 13.14% | 10.66% | 30 |
|  |  |  |  | *Pseudochrobactrum* | 0.56% | 2.15% | 25 |
|  |  |  |  | *Roseomonas* | 0.51% | 0.95% | 21 |
| Betaproteobacteria | 1.32% | 3.32% | 26 | *Burkholderia* | 0.76% | 3.03% | 11 |
| Gammaproteobacteria | 31.76% | 17.71% | 30 | *Erwinia* | 9.0% | 13.49% | 30 |
|  |  |  |  | *Pseudoxanthomonas* | 12.43% | 7.25% | 30 |
|  |  |  |  | *Xanthomonas* | 0.70% | 2.58% | 23 |
| Sphingobacteriia | 36.21% | 15.25% | 30 | *Olivibacter* | 10.62% | 10.41% | 29 |
|  |  |  |  | *Pedobacter* | 6.80% | 8.23% | 28 |
|  |  |  |  | Unknown Sphingobacteriia | 4.55% | 9.59% | 22 |
|  |  |  |  | *Sphingobacterium* | 13.56% | 12.83% | 29 |

Supplementary Table 2. Mean abundance and standard deviation of fungal and bacterial taxa in field nests by order and species with number of nests detected.

Supplementary Table 3. Mean abundance and standard deviation of fungal and bacterial taxa in laboratory nests by order and species with number of nests detected.

| **Fungi** | | | | | | | |
| --- | --- | --- | --- | --- | --- | --- | --- |
| ***Order*** | ***mean*** | ***SD*** | ***N*** | ***Species*** | ***mean*** | ***SD*** | ***N*** |
| Eurotiales | 0.58% | 5.65% | 43 |  |  |  |  |
| Ophiostomatales | 45.70% | 40.86% | 148 | *Raffaelea canadensis* | 6.67% | 15.49% | 144 |
|  |  |  |  | *Raffaelea sulphurea* | 38.61% | 38.11% | 147 |
| Sordariales | 52.96% | 41.29& | 148 | *Chaetomium globosum* | 52.16% | 41.33% | 148 |
| **Bacteria** | | | | | | | |
| ***Class*** | ***mean*** | ***SD*** | ***N*** | ***Genus*** | ***mean*** | ***SD*** | ***N*** |
| Actinobacteriia | 1.08% | 1.60% | 65 |  |  |  |  |
| Alphaproteobacteria | 1.73% | 11.56% | 72 | *Ochrobactrum* | 1.64% | 11.57% | 45 |
| Gammaproteobacteria | 97.13% | 11.56% | 89 | *Erwinia* | 7.56% | 21.72% | 80 |
|  |  |  |  | *Pantoea* | 0.81% | 7.35% | 42 |
|  |  |  |  | *Pseudoxanthomonas* | 83.32% | 30.65% | 89 |
|  |  |  |  | *Yersinia* | 5.18% | 20.20% | 43 |

**References**

Cao, Q., Sun, X., Rajesh, K., Chalasani, N., Gelow, K., Katz, B., et al. (2021). Effects of Rare Microbiome Taxa Filtering on Statistical Analysis. *Front. Microbiol.* 11, 3203. doi: 10.3389/FMICB.2020.607325/BIBTEX.

Davis, N. M., Proctor, Di. M., Holmes, S. P., Relman, D. A., and Callahan, B. J. (2018). Simple statistical identification and removal of contaminant sequences in marker-gene and metagenomics data. *Microbiome* 6. doi: 10.1186/S40168-018-0605-2.

Diehl, J. M. C., Kowallik, V., Keller, A., and Biedermann, P. H. W. (2022). First experimental evidence for active farming in ambrosia beetles and strong heredity of garden microbiomes. *Proc. R. Soc. B Biol. Sci.* 289. doi: 10.1098/rspb.2022.1458.

Nuotclà, J. A., Diehl, J. M. C., and Taborsky, M. (2021). Habitat quality determines dispersal decisions and fitness in a beetle – fungus mutualism. *Front. Ecol. Evol.* 9, 1–15. doi: 10.3389/fevo.2021.602672.
